# Supramolecular chirality transformation driven by monodentate ligand binding to a coordinatively unsaturated self-assembly based on *C*_3_-symmetric ligands†
†Electronic supplementary information (ESI) available: Additional experimental details and additional data. See DOI: 10.1039/c9sc00399a


**DOI:** 10.1039/c9sc00399a

**Published:** 2019-03-05

**Authors:** Yuki Imai, Junpei Yuasa

**Affiliations:** a Department of Applied Chemistry , Tokyo University of Science , 1-3, Kagurazaka, Shinjuku , Tokyo 162-8601 , Japan . Email: yuasaj@rs.tus.ac.jp

## Abstract

Monodentate ligand binding is facilitated by supramolecular chirality transformations from propeller-shaped chirality into single-twist chirality by altering the self-assembly of *C*_3_-symmetric chiral ligands. The *C*_3_-symmetric chiral ligands (**Im*^R^*_3_Bz** and **Im*^S^*_3_Bz**) contain three chiral imidazole side arms (Im^*R*^ and Im^*S*^) at the 1,3,5-positions of a central benzene ring. Upon coordination to zinc ions (Zn^2+^), which have a tetrahedral coordination preference, the *C*_3_-symmetric chiral ligands assemble, in a stepwise manner, into a propeller-shaped assembly with a general formula (**Im^(^*^R^*^or^*^S^*^)^_3_Bz**)_4_(Zn^2+^)_3_. In this structure each Zn^2+^ ion coordinates to the three individual imidazole side arms. The resulting assembly is formally coordinatively unsaturated (coordination number, *n* = 3) and capable of accepting monodentate co-ligands (imidazole: ImH_2_) to afford a coordinatively saturated assembly [(ImH_2_)_3_(**Im*^R^*_3_Bz**)_4_(Zn^2+^)_3_]. The preformed propeller-shaped chirality is preserved during this transformation. However, an excess of the monodentate co-ligand (ImH_2_/Zn^2+^ molar ratio of ∼1.7) alters the propeller-shaped assembly into a stacked dimer assembly [(ImH_2_)_*m*_(**Im*^R^*_3_Bz**)_2_(Zn^2+^)_3_] (*m* = 4–6) with single-twist chirality. This switch alters the degree of enhancement and the circular dichroism (CD) pattern, suggesting a structural transition into a chiral object with a different shape. This architectural chirality transformation presents a new approach to forming dynamic coordination-assemblies, which have transformable geometric chiral structures.

## Abstract 2

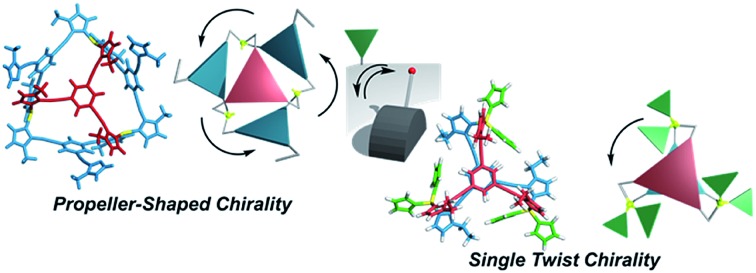
A supramolecular chirality transition driven by monodentate ligand binding, the present strategy shows promise for the rational design of dynamic coordination chirality capable of alternating between chiral objects of different shapes driven by a specific external stimulus.

## Introduction

Topology plays an important role in determining the properties of self-assembled structures at various hierarchical levels from the molecular to supramolecular scales. Among features that determine structure and function relationships, chirality mainly determines physical and morphological properties.^1^ Nature's topology is dynamic and transforms under the influence of certain stimuli.^2^–^4^ However, in the field of coordination assembly, supramolecular chirality is considered to be a static feature. In most cases, building blocks are rigid and inflexible, fixing the directionality of metal–ligand interactions, which is a requirement for predetermined chirality in discrete metallosupramolecular architectures.^1^,^5^ Conversely, the concept of dynamic assembly has drawn attention to the benefits of dynamic structures that can react to certain stimuli and change their behavior.^6^–^23^ For example, dynamic assembly has made it possible to design supramolecular architectures with “chirality inversion” through changes to an explicit chiral shape (*e.g.*, helices), which is successfully reflected by chiroptical inversion, such as circular dichroism (CD) and circularly polarized luminescence (CPL) spectra.^7^–^14^ Besides these successful achievements, few cases of dynamic topology alteration have been reported and they have mostly concerned achiral to achiral transformations in two-component systems consisting of only one ligand component and one metal ion component.^18^–^21^ In such cases, an appropriate ratio of the building components drives alternative shapes in the output architecture. Stimuli-responsive or “smart” coordination assemblies^22^,^23^ have two competing features that must be balanced: the assembly should have metastability to enable acceptance of a third component (*e.g.*, co-ligand), which induces the stimuli-responsive transformations. The system should also have sufficient thermodynamic stability to selectively form the target assembly in solution prior to any stimuli. It remains challenging to identify systems where both these properties are achieved.

**Scheme 1:**
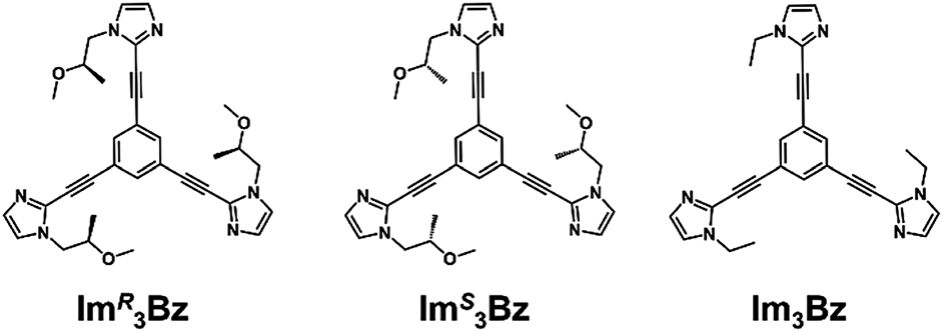
Chemical structures of *C*_3_-symmetric ligands studied here.

**Scheme 2:**
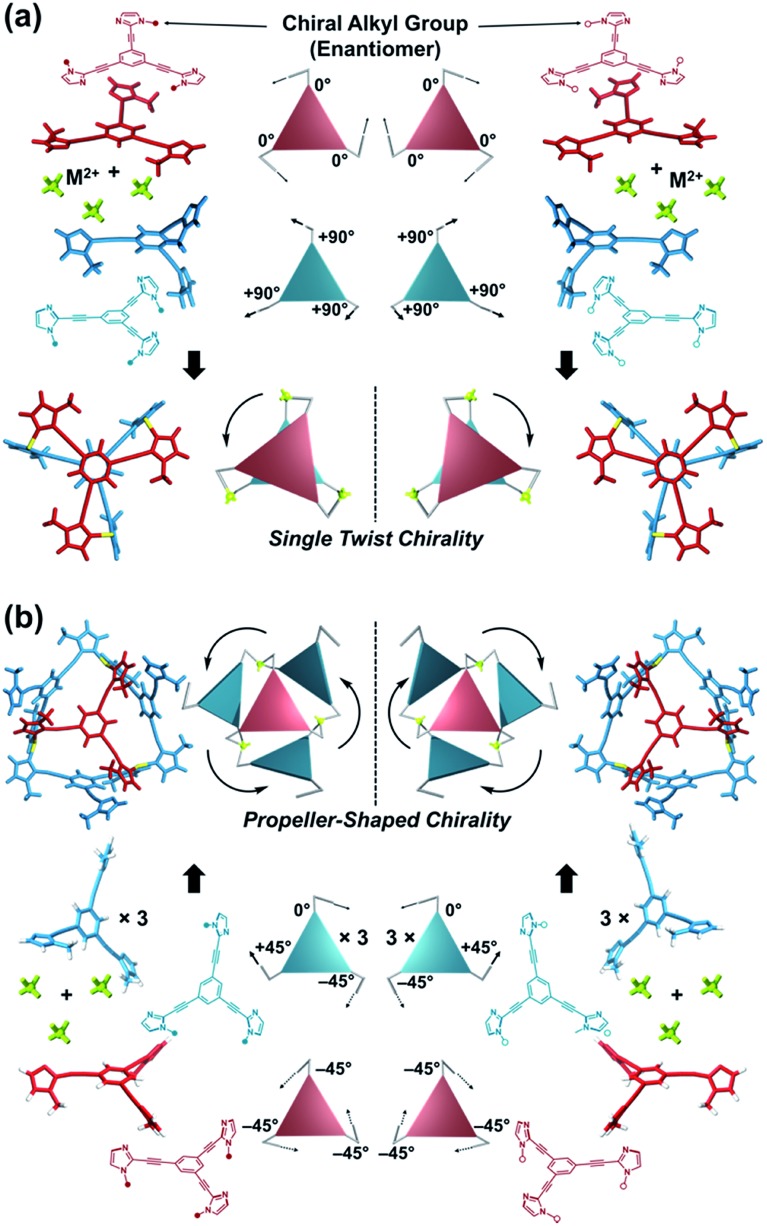
(a) Supramolecular architecture with single twist chirality formed from the two *C*_3_-symmetric ligands clipped by three metal ions with a tetrahedral coordination preference. It should be noted that the suggested L_2_M_3_ complex was not formed in the present system in the absence of a monodentate co-ligand. (b) Supramolecular architecture with propeller-shaped chirality formed from the three *C*_3_-symmetric ligands clipped by three metal ions with a tetrahedral coordination preference.

Here, we propose a flexible ligand approach to address supramolecular chirality transformations driven by monodentate ligand binding. We have designed new *C*_3_-symmetric ligands (**Im*^R^*_3_Bz**, **Im*^S^*_3_Bz**, and **Im_3_Bz**) containing three imidazole side arms connected to a central benzene ring at the 1,3,5-positions through ethynyl spacers (Scheme 1). The present *C*_3_-symmetric ligands show intrinsic rotational flexibility around the ethynyl axes but maintain conjugated aromatic units, resulting in structural diversity in terms of the dihedral angle (*θ*) between the imidazole and the central benzene rings (Scheme 2). Most *C*_3_-symmetric ligands containing ethynyl spacers have been designed with pyridine rings to make the direction of the metal–ligand interactions constant irrespective of the ligand conformation prior to self-assembly.^24^,^25^ This diversity in ligand chirality suggests potential to access a range of topologies with different chiral shapes through a coordination process we term “metal-ion clipping”.^21^ Among the types of supramolecular chirality accessible in this coordination assembly, the simplest should be a stacked dimer with single-twist chirality (Scheme 2 from top to middle).21c,^26^,^27^ This structure forms from two different *C*_3_-symmetric conformers (*θ* = +90°, +90°, +90°; *θ* = 0°, 0°, 0°) clipped by three metal ions with a tetrahedral coordination preference. Propeller-shaped chirality is another probable architecture,^28^ which forms from a *C*_3_-symmetric conformer (*θ* = –45°, –45°, –45°) with three non-symmetrical conformers (*θ* = –45°, +45°, 0°) clipped by three metal ions (Scheme 2 from bottom to middle). We reveal that the *C*_3_-symmetric ligands selectively assemble into a propeller-shaped chiral assembly upon coordination to zinc ions (Zn^2+^). The resulting propeller-shaped assembly [(**Im^(^*^R^*^or^*^S^*^)^_3_Bz**)_4_(Zn^2+^)_3_] is formally coordinatively unsaturated (coordination number, *n* = 3), and undergoes coordination of monodentate co-ligands (imidazole: ImH_2_) to afford a coordinatively saturated assembly [(ImH_2_)_3_(**Im^(^*^R^*^or^*^S^*^)^_3_Bz**)_4_(Zn^2+^)_3_]. The resulting coordinatively saturated assembly maintains the preformed propeller-shaped chirality but transforms into a stacked dimer assembly [(ImH_2_)_*m*_(**Im^(^*^R^*^or^*^S^*^)^_3_Bz**)_2_(Zn^2+^)_3_] (*m* = 4–6) with single-twist chirality at a certain excess of monodentate co-ligands (ImH_2_/Zn^2+^ ratio at approximately 1.7). This change considerably enhances and modifies the CD signals, suggesting that the structure transitions into different chiral objects with different shapes. The present system seems to be completely different from the past examples of the structural conversion associated with chiroptical inversion by external stimuli. Our findings will pave the way for rational design of dynamic and flexible coordination chirality, which can transform between different chiral shapes under stimuli.

## Results and discussion

### Chiral assembly formation of *C*_3_-symmetric chiral ligands upon coordination to zinc ions

**Fig. 1:**
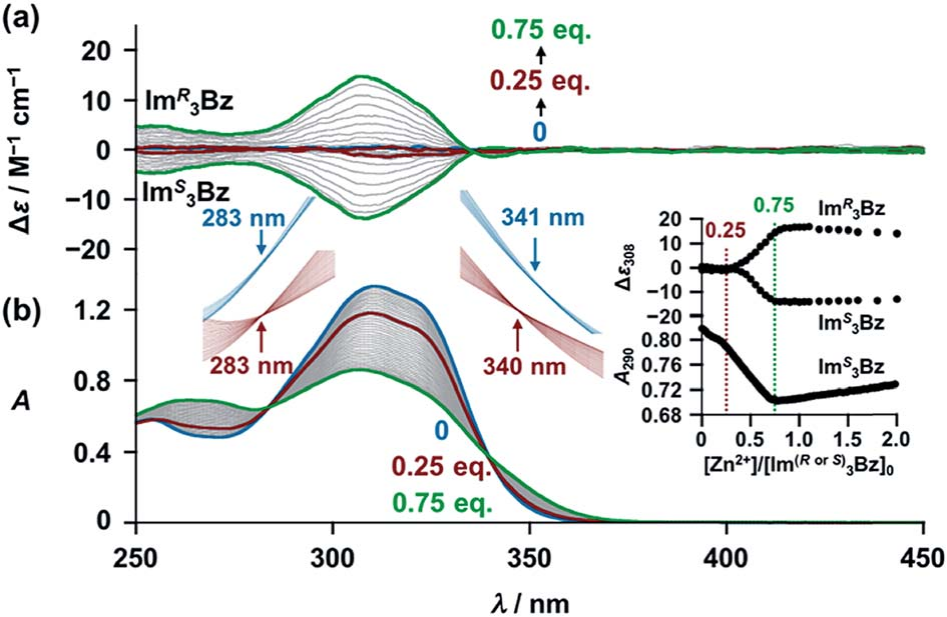
(a) CD spectra of **Im*^R^*_3_Bz** and **Im*^S^*_3_Bz** (concentrations: 2.0 × 10^–5^ M) in the presence of Zn^2+^ [0 (blue), 5.0 × 10^–6^ (red) and 1.5 × 10^–5^ M (green)] in acetonitrile at 298 K, where Δ*ε* is calculated based on the ligand concentration (2.0 × 10^–5^ M). (b) Corresponding UV/vis absorption spectral changes observed in the titration of **Im*^S^*_3_Bz** by Zn^2+^. Inset: plots of Δ*ε* at 308 nm absorbance at 290 nm *versus* [Zn^2+^]/[**Im^(^*^R^*^or^*^S^*^)^_3_Bz**]_0_. The magnified spectra around the isosbestic points (blue lines: [Zn^2+^] = 0–5.0 × 10^–6^ M; red lines: 5.0 × 10^–6^ M to 1.5 × 10^–5^ M). For clarity reasons two step spectral changes are separated vertically.

**Fig. 2:**
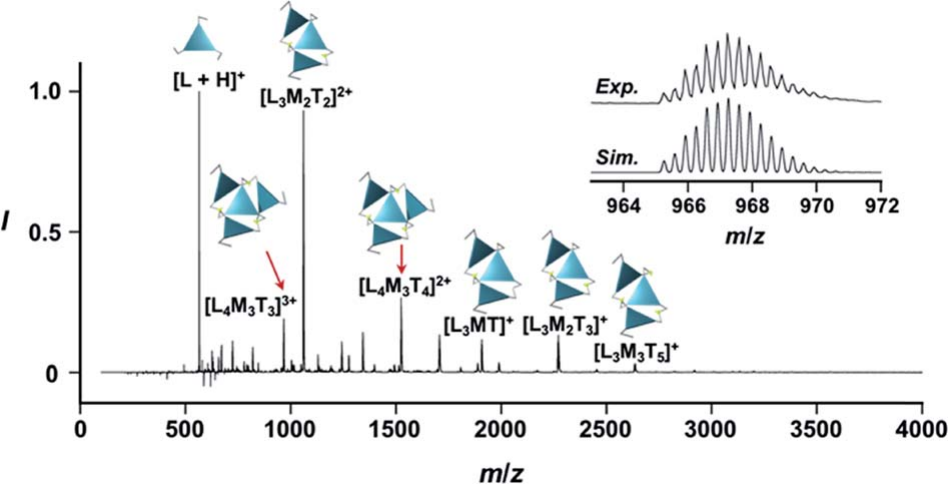
Positive ESI-MS spectrum of **Im*^S^*_3_Bz** (normalized by its most intense fragment at *m*/*z* = 564.3 from the free ligand) in acetonitrile (2.0 × 10^–3^ M) in the presence of Zn^2+^ (1.6 × 10^–3^ M). Inset: isotopically resolved signals at *m*/*z* = 965.3 and the calculated isotopic distributions for [(**Im*^S^*_3_Bz**)_4_(Zn^2+^)_3_(OSO_2_CF_3_)_3_]^3+^. Objects correspond to the mass peak assignment (L: **Im*^S^*_3_Bz**, M: Zn^2+^, T: OSO_2_CF_3_^–^).

**Fig. 3:**
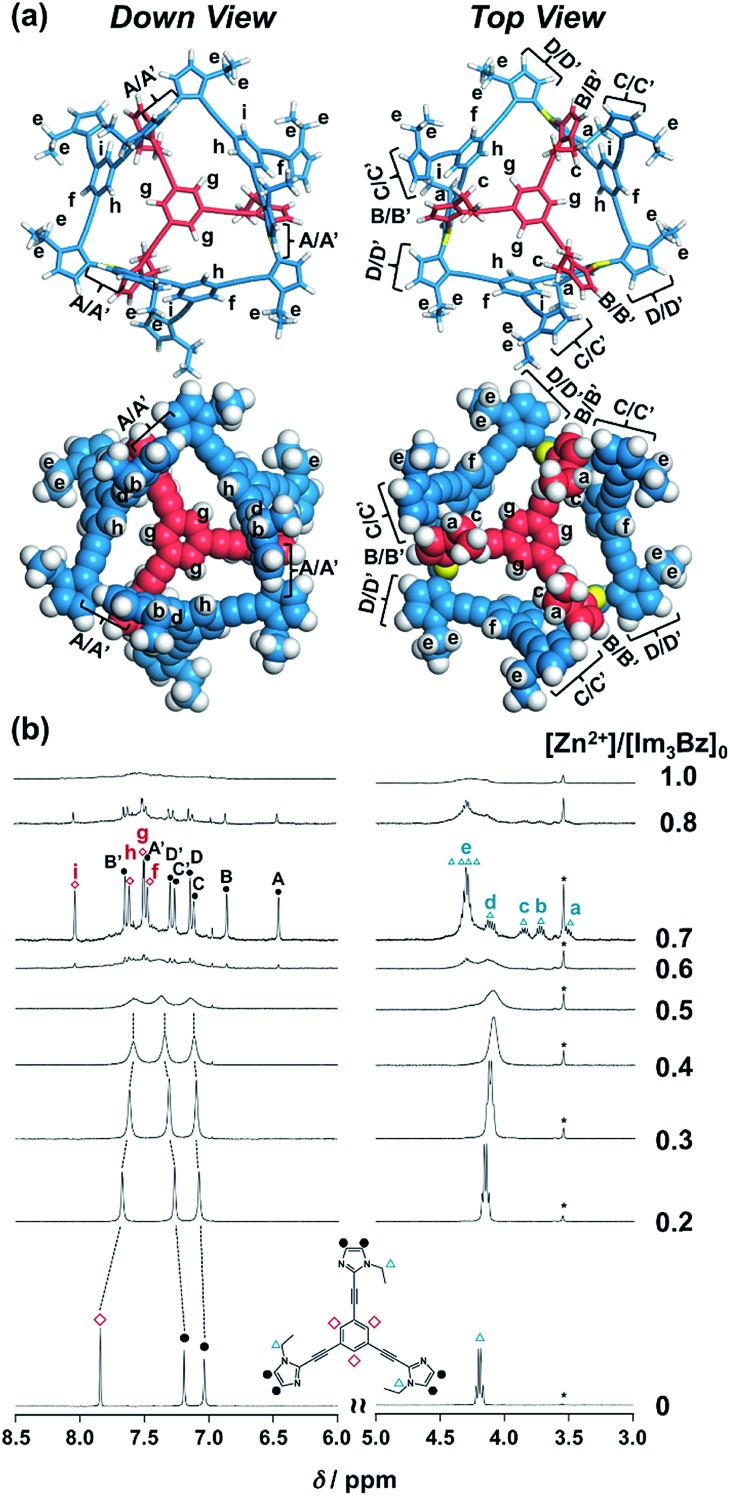
(a) Solution structure and NMR signal assignment of (**Im_3_Bz**)_4_(Zn^2+^)_3_. (b) Stacked ^1^H NMR spectra of **Im_3_Bz** (3.3 × 10^–3^ M) in the presence of Zn^2+^ (0–3.3 × 10^–3^ M) in CD_3_CN at 298 K. The small peak annotated by an asterisk remains unchanged during the titration. Symbols correspond to those in the chemical structure of **Im_3_Bz**. Alphabet pairs (A/A′, B/B′, C/C′, and D/D′) labeling closed circles indicate vicinal protons at the imidazole ring.

**Scheme 3:**
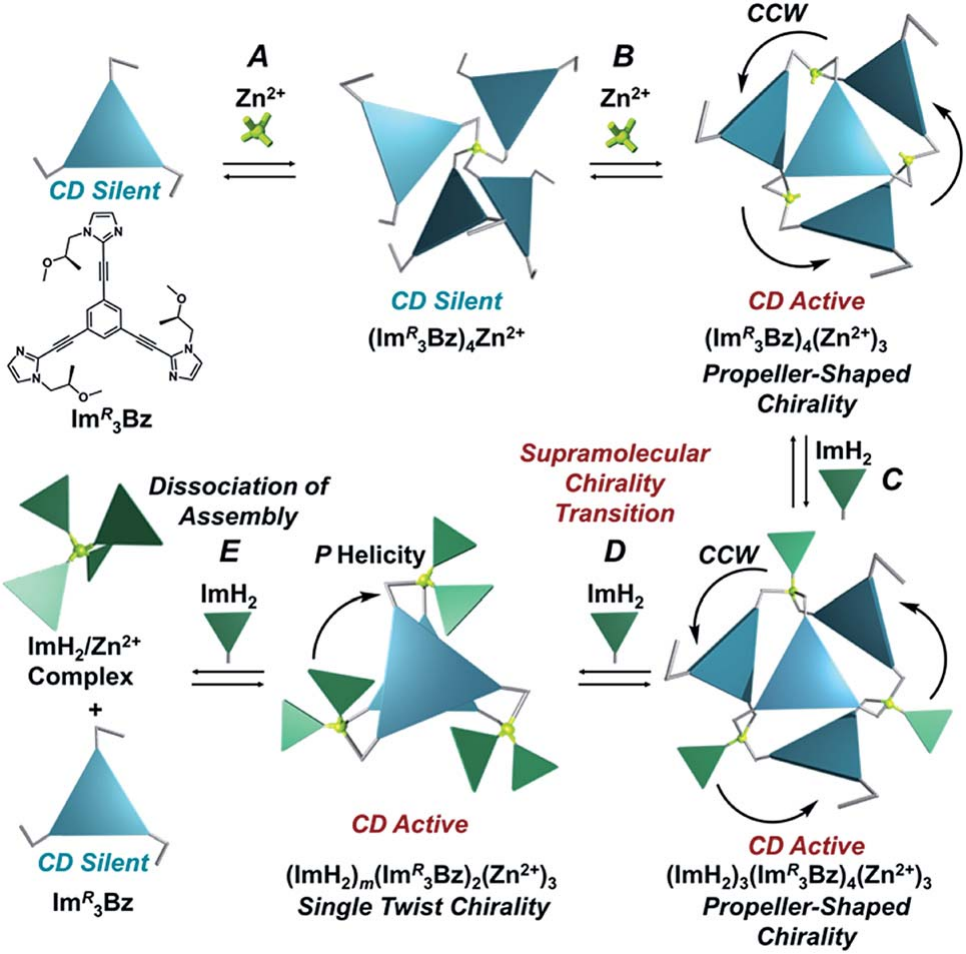
Assembly formation of **Im*^R^*_3_Bz** with Zn^2+^ and ImH_2_.

New *C*_3_-symmetric ligands (**Im*^R^*_3_Bz**, **Im*^S^*_3_Bz**, and **Im_3_Bz**) were synthesized by Sonogashira coupling between the corresponding 2-iodoimidazoles and the 1,3,5-tris(ethynylbenzene) linker (see the ESI†). We used circular dichroism (CD) to investigate the chiral assembly of the *C*_3_-symmetric ligands upon coordination to zinc ions (Zn^2+^). Although the *C*_3_-symmetric chiral ligands contain chiral alkyl side chains, neither **Im*^R^*_3_Bz** nor **Im*^S^*_3_Bz** show a CD signal in the absence of Zn^2+^ (Fig. 1a, blue lines), because their chiral alkyl side chains are separated from the central chromophore unit (Scheme 1). However, the *C*_3_-symmetric chiral ligands exhibit a unique CD signal pattern once they assemble into geometrically chiral objects with Zn^2+^ (Fig. 1a, green lines, *vide infra*).21*c* Hence, the CD signals act as a probe for geometric changes occurring in the solution assembly process. These findings are further supported by analysis of NMR titration and ESI mass spectrometry results (Fig. 2 and 3, *vide infra*). The CD titration experiments were performed in acetonitrile solutions containing a constant concentration of **Im*^R^*_3_Bz** or **Im*^S^*_3_Bz** ([**Im^(^*^R^*^or^*^S^*^)^_3_Bz**]_0_ = 2.0 × 10^–5^ M) by changing the concentration of the Zn^2+^ ions (counter anion: OSO_2_CF_3_^–^). These results were compared with those from UV/vis titration experiments conducted under the same conditions (Fig. 1b). At a molar ratio of [Zn^2+^]/[**Im^(^*^R^*^or^*^S^*^)^_3_Bz**]_0_ = 0.25, Zn^2+^ induced no appreciable CD signal in the region of the π–π* transition (*λ* < 360 nm) of the *C*_3_-symmetric ligands (Fig. 1a, red lines); however, the absorption changed noticeably with isosbestic points at 283 and 341 nm (Fig. 1b, blue line to red line). The absorption spectral changes suggest conversion of **Im^(^*^R^*^or^*^S^*^)^_3_Bz** into a Zn^2+^ complex, which has no CD activity (Scheme 3A). However, as the molar ratio of [Zn^2+^]/[**Im^(^*^R^*^or^*^S^*^)^_3_Bz**]_0_ increased beyond 0.25, **Im*^R^*_3_Bz** and **Im*^S^*_3_Bz** exhibited positive and negative Cotton effects, respectively. Almost complete mirror-image CD signals can be seen between the enantiomers (Fig. 1a, green lines). Together with the considerable changes in the CD spectra, **Im*^S^*_3_Bz** showed changes in its absorption spectra with isosbestic points at 283 and 340 nm (Fig. 1b, red to green line).^30^ These results suggest a change of the CD-silent Zn^2+^-complex into a geometrically chiral Zn^2+^-assembly with CD activity (Scheme 3B). The CD intensity (Δ*ε*) at 308 nm and the absorbance at 290 nm are plotted against the molar ratio ([Zn^2+^]/[**Im^(^*^R^*^or^*^S^*^)^_3_Bz**]_0_) (inset of Fig. 1). This plot illustrates the stoichiometry of the initially formed CD-silent Zn^2+^-complex and the subsequently formed Zn^2+^-assembly with CD activity. A clear break can be seen at [Zn^2+^]/[**Im^(^*^R^*^or^*^S^*^)^_3_Bz**]_0_ = 0.25 in each case, indicating a 4 : 1 complex of **Im^(^*^R^*^or^*^S^*^)^_3_Bz** and Zn^2+^ [(**Im^(^*^R^*^or^*^S^*^)^_3_Bz**)_4_Zn^2+^], which conforms to the most common coordination number of Zn^2+^-complexes (*n* = 4). The 4 : 1 complex was assigned by high-resolution electrospray injection mass spectrometry (HR ESI-MS) (positive): *m*/*z* calcd. [(**Im*^S^*_3_Bz**)_4_(Zn^2+^)_1_]^2+^, 1160.53435; found 1160.53384.^29^ Saturation of the titration curves occurred at [Zn^2+^]/[**Im^(^*^R^*^or^*^S^*^)^_3_Bz**]_0_ = 0.75 (inset of Fig. 1), which corresponds to 4 : 3 binding stoichiometry for **Im^(^*^R^*^or^*^S^*^)^_3_Bz** and Zn^2+^. The most probable assembly is a 4 : 3 assembly [(**Im^(R or S)^_3_Bz**)_4_(Zn^2+^)_3_] with the lowest global complexity (GC = 4 + 3 = 7). To confirm the stoichiometry and the global complexity of the Zn^2+^-assembly in solution, we measured the ESI mass of **Im*^S^*_3_Bz** in the presence of 0.8 equivalents of Zn(OSO_2_CF_3_)_2_ in acetonitrile (Fig. 2). The proposed (**Im*^S^*_3_Bz**)_4_(Zn^2+^)_3_ was successfully detected as [(**Im*^S^*_3_Bz**)_4_(Zn^2+^)_3_(OSO_2_CF_3_)_3_]^3+^ and [(**Im*^S^*_3_Bz**)_4_(Zn^2+^)_3_(OSO_2_CF_3_)_4_]^2+^, in which the isotopic distribution of [(**Im*^S^*_3_Bz**)_4_(Zn^2+^)_3_(OSO_2_CF_3_)_3_]^3+^ agreed well with the calculated spectrum (Fig. 2 inset). We identified several fragment ion peaks, including [(**Im*^S^*_3_Bz**)_3_(Zn^2+^)_3_(OSO_2_CF_3_^–^)_5_]^+^ (*m*/*z* = 2629.4) and [(**Im*^S^*_3_Bz**)_3_(Zn^2+^)_2_(OSO_2_CF_3_^–^)_2_]^2+^ (*m*/*z* = 2118.6). Thus (**Im*^S^*_3_Bz**)_4_(Zn^2+^)_3_ contains labile ligand units (*vide infra*).

On the basis of the above analysis, we propose a propeller-shaped structure (Fig. 3a) for the (**Im^(^*^R^*^or^*^S^*^)^_3_Bz**)_4_(Zn^2+^)_3_ assembly, which is the most symmetrical geometry of the Zn^2+^-assemblies with GC = 7 and a stoichiometry of [**Im^(^*^R^*^or^*^S^*^)^_3_Bz**] : [Zn^2+^] = 4 : 3. Single-crystal X-ray diffraction analysis provides definitive structural information in the solid state. However, the propeller-shaped structure is formally coordinatively unsaturated (coordination number, *n* = 3) in solution, and unfortunately suitable crystals of the self-assembly could not be obtained. Hence, we verified the proposed structure by systematic NMR spectroscopic titration analysis.^21^,^31^ Upon titration of Zn(OSO_2_CF_3_)_2_ into **Im*^S^*_3_Bz** in acetonitrile-*d*_3_ (CD_3_CN) broadening of the ^1^H NMR signals occurred because of rapid exchange, *i.e.*, dissociation and reassociation of the (**Im*^S^*_3_Bz**)_4_(Zn^2+^)_3_ assembly on the NMR timescale (Fig. S1†). We found that the NMR line broadening was prevented by modifying the side arms of the *C*_3_-symmetric ligands (Scheme 1), when the chiral chains were replaced by ethyl groups (*vide infra*, Fig. 3b). We note that the **Im_3_Bz** ligand exhibited absorption titration and ESI mass spectrometric results (Fig. S2 and S3†) similar to those for **Im^(^*^R^*^or^*^S^*^)^_3_Bz** (Fig. 1 and 2), which confirmed that **Im_3_Bz** also forms a 4 : 3 assembly [(**Im_3_Bz**)_4_(Zn^2+^)_3_] in a stepwise manner (as Scheme 3A and B). During the titration of **Im_3_Bz** with Zn^2+^ at [Zn^2+^]/[**Im_3_Bz**]_0_ molar ratios in the range of 0–0.6, the aromatic protons of the imidazole side arms and the central benzene rings gradually shifted and broadened (Fig. 3b). The initially observed NMR peak shift at [Zn^2+^]/[**Im_3_Bz**]_0_ molar ratios in the range of 0–0.4 is attributed to the formation of a 4 : 1 complex [(**Im_3_Bz**)_4_Zn^2+^]. Conversely, the NMR line broadening likely originated from rapid exchange between the resulting 4 : 1 complex [(**Im_3_Bz**)_4_Zn^2+^] and the subsequently formed 4 : 3 self-assembled [(**Im_3_Bz**)_4_(Zn^2+^)_3_]. However, clear NMR signals with a total of 12 aromatic protons were resolved upon addition of a certain amount (0.7 equivalents) of Zn^2+^ (Fig. 3b left).^32^ The preformed 4 : 1 complex was almost completely converted into the 4 : 3 self-assembly at a molar ratio close to [Zn^2+^]/[**Im_3_Bz**]_0_ = 0.75. On the basis of COSY correlations (Fig. S5†), we separated the 12 observed resonances into 4 resonances originating from the central benzene protons (Fig. 3b red squares, f, g, h, and i) and 8 other resonances corresponding to the imidazole (aromatic) protons (Fig. 3b closed circles, A–D and A′–D′). Among the central benzene protons (f, g, h, and i), we observed the ^1^H, ^1^H COSY correlations between the signals “f”, “h”, and “i” (Fig. S5†). Therefore, these signals come from neighboring protons that are directly bound to the same central benzene ring, but located in different chemical environments. Thus (**Im_3_Bz**)_4_(Zn^2+^)_3_ has **Im_3_Bz** ligands that lose the *C*_3_ symmetry of the original ligand upon self-assembly (blue colored molecules in Fig. 3a). Conversely, the benzene proton “g” has no ^1^H, ^1^H COSY correlation with other benzene protons (f, h, and i, see Fig. S5†), and therefore (**Im_3_Bz**)_4_(Zn^2+^)_3_ contains a central **Im_3_Bz** ligand that preserves the original ligand *C*_3_ symmetry (red colored molecule in Fig. 3a). On the basis of these results, we present the solution structure of (**Im_3_Bz**)_4_(Zn^2+^)_3_ (Fig. 3a), consisting of a central *C*_3_-symmetric ligand (red colored molecule) surrounded by three non-symmetrical conformers (blue colored molecules). Consequently, the proposed propeller-shaped assembly gives a total of four benzene protons with an integration ratio of 1 : 1 : 1 : 1 (3H : 3H : 3H : 3H) in its NMR spectrum. One of these protons (g) comes from the central *C*_3_-symmetric ligand (red colored molecule) and the other three protons (f, h, and i) come from the three ligands (blue colored molecules). In this manner, NMR shows a total of 8 resonances for imidazole (aromatic) protons with the same integration ratio (3H amount for each signal), which is consistent with the experimentally observed NMR signals arising from the four pairs of vicinal protons at the imidazole rings (Fig. 3b, A/A′, B/B′, C/C′, and D/D′). The ethyl proton signals are likely caused by the imidazole side chains as much simpler probes, which support the proposed propeller-shaped structure. In the presence of 0.7 equivalents of Zn^2+^, the original quartet signal at 4.17 ppm splits into a double quartet signals with an integration ratio of 1 : 1 : 1 : 1 (Fig. 3b, blue triangles, a–d; 3H for each signal) and an intense overlapped signal consisting of 12 protons (e). The double quartet signals (a–d) suggest that the two CH_2_ protons (connected to the same CH_2_ carbon atom) are in different chemical environments owing to restricted rotation of the ethyl side chains upon formation of the propeller-shaped assembly. In fact, we observed ^1^H, ^1^H COSY correlations between “a” and “c” and between “b” and “d” (Fig. S6†). Therefore, these proton pairs must be connected to the same CH_2_ carbon atom. Among the four double quartet signals (a–d), the proton pairs “a” and “c” are assignable to the CH_2_ protons of the central *C*_3_-symmetric ligand (red colored molecule in Fig. 3a). These protons are shielded by the neighboring imidazole rings of the three propeller-shaped ligands (blue colored molecules). This steric configuration should restrict the rotation of the ethyl side chains in the central *C*_3_-symmetric ligand (red colored molecule). Conversely, the other proton pairs (b and d) are attributed to the CH_2_ protons of the side chains of the other three **Im_3_Bz** (blue colored molecules) at the bottom position. Consequently, the intense overlapping signal (e) corresponds to the other ethyl side chains of the three propeller-shaped ligands (blue colored molecules) at the top position in similar chemical environments (see the top view in Fig. 3a). The NMR line broadening above 0.8 equiv. of Zn^2+^ (Fig. 3b) indicates that an excess of Zn^2+^ ions promoted rapid dissociation and reassociation processes of (**Im_3_Bz**)_4_(Zn^2+^)_3_. On the basis of these findings, we conclude that the propeller-shaped structure (Fig. 3a) is the most probable main species in solution.^33^

### Supramolecular chirality transformation driven by monodentate ligand binding

**Fig. 4:**
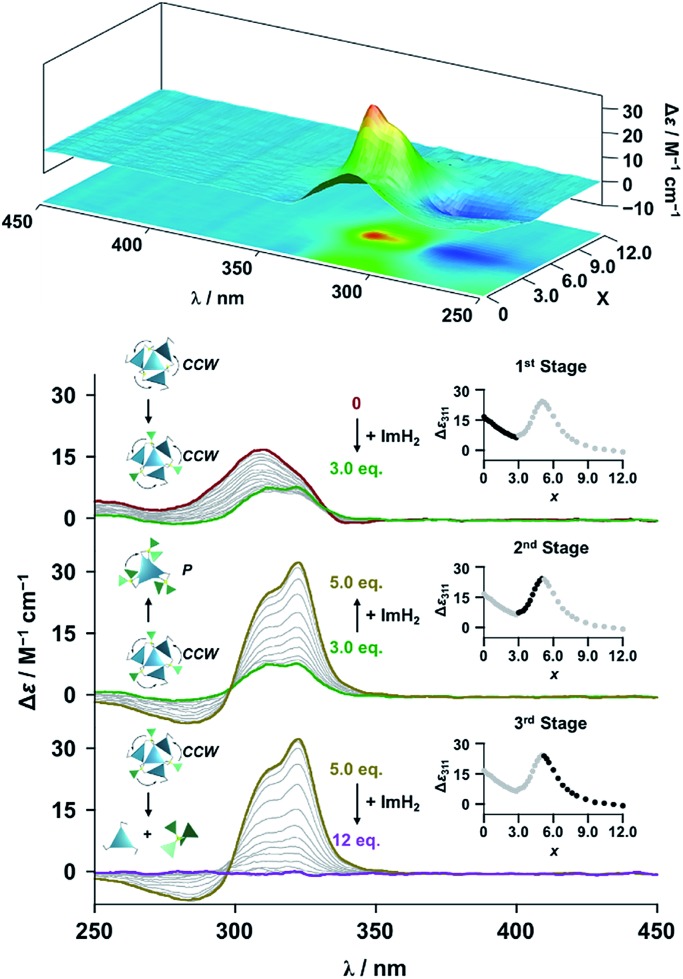
CD spectral changes observed upon titration of an acetonitrile solution of **Im*^R^*_3_Bz** (2.0 × 10^–5^ M) containing Zn^2+^ (2.0 × 10^–5^ M) with ImH_2_ (0–6.0 × 10^–5^ M, 0: red line, 1.5 × 10^–5^ M: green line, 2.5 × 10^–5^ M: yellow line, and 6.0 × 10^–5^ M: purple line), where Δ*ε* is calculated based on the ligand concentration (2.0 × 10^–5^ M). Top panel shows the corresponding stacked plot for CD titration. Insets: plot of Δ*ε* at 311 nm *vs.* the molar ratio (*x* = [ImH_2_]/[(**Im*^R^*_3_Bz**)_4_(Zn^2+^)_3_]_0_).

**Fig. 5:**
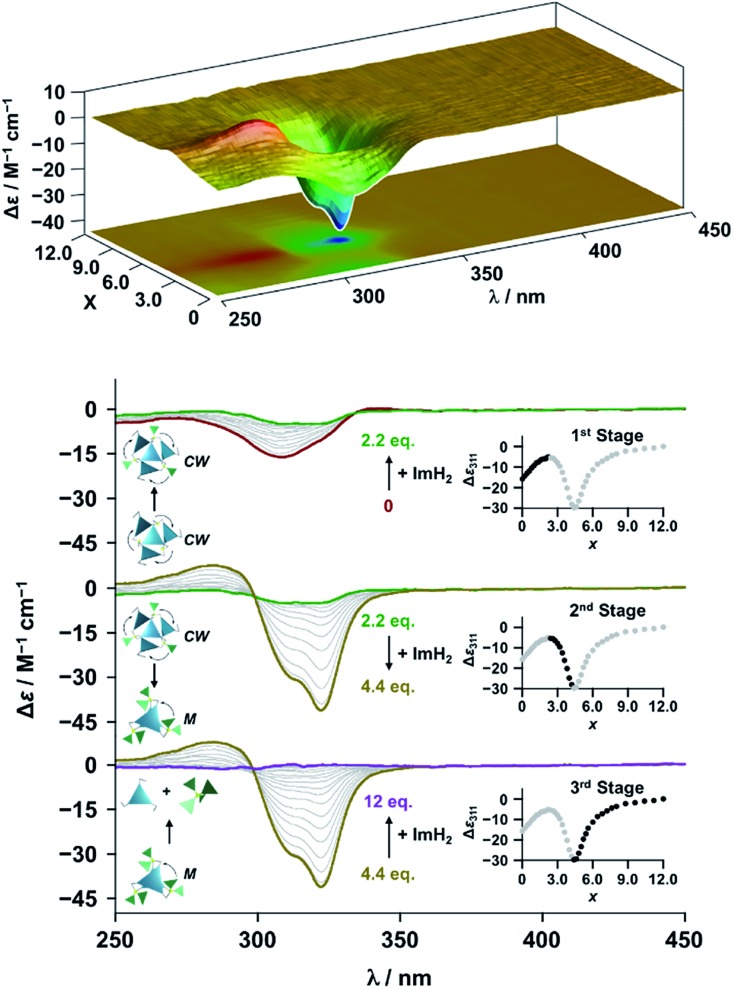
CD spectral changes observed upon titration of an acetonitrile solution of **Im*^S^*_3_Bz** (2.0 × 10^–5^ M) containing Zn^2+^ (2.0 × 10^–5^ M) with ImH_2_ (0–6.0 × 10^–5^ M, 0: red line, 1.5 × 10^–5^ M: green line, 2.2 × 10^–5^ M: yellow line, and 6.0 × 10^–5^ M: purple line), where Δ*ε* is calculated based on the ligand concentration (2.0 × 10^–5^ M). Top panel shows the corresponding stacked plot for CD titration. Insets: plot of Δ*ε* at 311 nm *vs.* the molar ratio (*x* = [ImH_2_]/[(**Im*^S^*_3_Bz**)_4_(Zn^2+^)_3_]_0_).

**Fig. 6:**
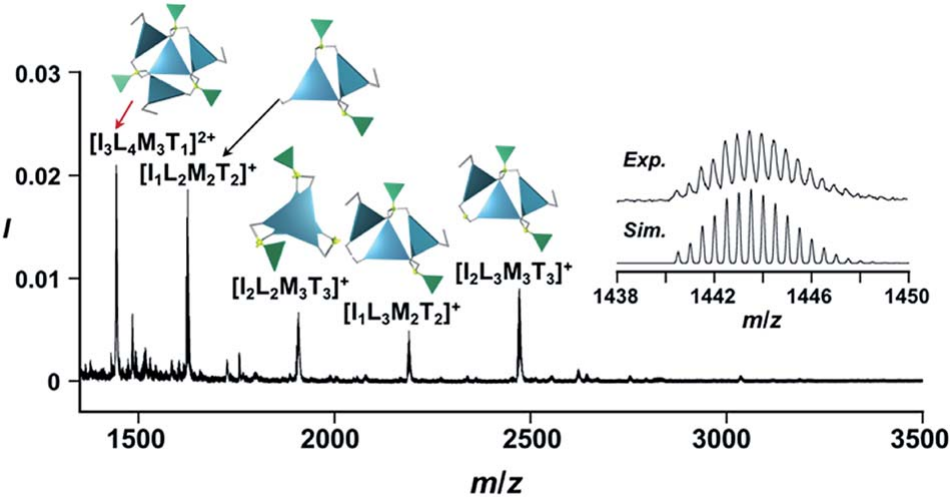
Positive ESI-MS spectrum of **Im*^S^*_3_Bz** (normalized by its most intense fragment at *m*/*z* = 564.3 from the free ligand) in acetonitrile (2.2 × 10^–3^ M) in the presence of Zn^2+^ (1.7 × 10^–3^ M) and ImH_2_ (1.7 × 10^–3^ M). Inset: isotopically resolved signals at *m*/*z* = 1440.5 and calculated isotopic distributions for [(ImH^–^)_3_(**Im*^S^*_3_Bz**)_4_(Zn^2+^)_3_(OSO_2_CF_3_)(CH_3_CN)_2_]^2+^. Objects correspond to the mass peak assignment (I: ImH^–^, L: **Im*^S^*_3_Bz**, M: Zn^2+^, T: OSO_2_CF_3_^–^). Mass spectrum of the whole region is given in Fig. S9.†

**Fig. 7:**
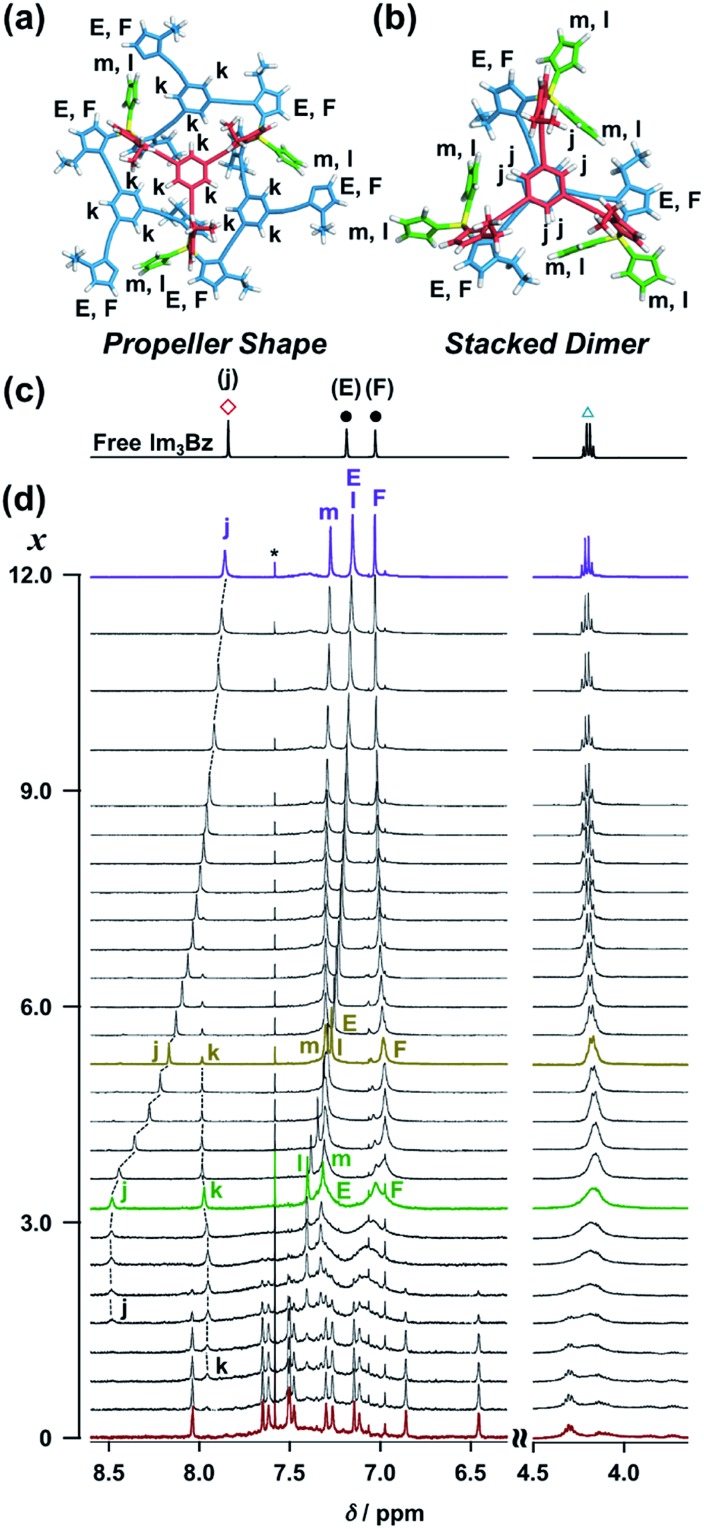
Energy minimized structures and NMR signal assignment of (a) (ImH_2_)_3_(**Im_3_Bz**)_4_(Zn^2+^)_3_ and (b) (ImH_2_)_6_(**Im_3_Bz**)_2_(Zn^2+^)_3_. (c) ^1^H NMR spectrum of free **Im_3_Bz** (1.8 × 10^–3^ M) in CD_3_CN, where symbols correspond to those in the chemical structure **Im_3_Bz** (Fig. 3). (d) Stacked ^1^H NMR spectra of **Im_3_Bz** (1.8 × 10^–3^ M) with Zn^2+^ (1.4 × 10^–3^ M) in the presence of ImH_2_ (0–5.4 × 10^–3^ M, 0: red line, 1.4 × 10^–3^ M: green line, 2.3 × 10^–3^ M: yellow line, and 5.4 × 10^–3^ M: purple line) in CD_3_CN at 298 K (*x* = [ImH_2_]/[(**Im_3_Bz**)_4_(Zn^2+^)_3_]_0_). Asterisk denotes chloroform.

The propeller-shaped assembly is formally coordinatively unsaturated (Fig. 3a), where each Zn^2+^ ion coordinates to the three individual imidazole side arms (coordination number, *n* = 3). On the basis of ESI mass spectrometric analysis in solution (Fig. 2, *vide supra*), their vacant sites are likely occupied by solvent molecules or counter anions (OSO_2_CF_3_^–^), which can be easily replaced by an appropriate monodentate co-ligand. This motivated us to investigate monodentate ligand binding (imidazole: ImH_2_) to the resulting coordinatively unsaturated assembly (Scheme 3C and E). This process was again monitored by CD spectroscopy (Fig. 4 and 5; UV/vis titration in Fig. S8†). Upon addition of 0–3.0 equivalents of ImH_2_ to the resulting (**Im^(^*^R^*^or^*^S^*^)^_3_Bz**)_4_(Zn^2+^)_3_ assembly in acetonitrile, the CD spectrum of the propeller-shaped assembly [(**Im^(^*^R^*^or^*^S^*^)^_3_Bz**)_4_(Zn^2+^)_3_] gradually disappeared (Fig. 4 and 5, red line to green line). We then found a sharp change of the signal with an enhancement of the CD intensity in the presence of 5.0 equivalents (ImH_2_/Zn^2+^ molar ratio: ∼1.7) of ImH_2_ (Fig. 4 and 5, green line to yellow line). The resulting CD signals almost disappeared upon further addition of 12 equivalents of ImH_2_ (Fig. 4 and 5, purple line), owing to dissociation of self-assemblies in the presence of excess ImH_2_, which yielded ImH_2_/Zn^2+^ complexes (Scheme 3E). We analyzed the observed stepwise process (Scheme 3C and E) by using titration plots (insets of Fig. 4 and 5) of Δ*ε* at 311 nm *vs.* the molar ratio (*x* = [ImH_2_]/[(**Im^(^*^R^*^or^*^S^*^)^_3_Bz**)_4_(Zn^2+^)_3_]_0_), where inflection points occurred at [ImH_2_]/[(**Im^(^*^R^*^or^*^S^*^)^_3_Bz**)_4_(Zn^2+^)_3_]_0_ = 3.0 and around 5.0. The initial inflection is responsible for binding of the monodentate co-ligand (ImH_2_) to the vacant sites of (**Im^(^*^R^*^or^*^S^*^)^_3_Bz**)_4_(Zn^2+^)_3_, yielding a coordinatively saturated (ImH_2_)_3_(**Im^(^*^R^*^or^*^S^*^)^_3_Bz**)_4_(Zn^2+^)_3_ assembly (Scheme 3C). The resulting CD pattern of (ImH_2_)_3_(**Im^(^*^R^*^or^*^S^*^)^_3_Bz**)_4_(Zn^2+^)_3_ resembles the original CD profile owing to the propeller-shaped assembly despite the subtle red shift (Fig. 4 and 5, red line *vs.* green line), indicating that the resulting (ImH_2_)_3_(**Im^(^*^R^*^or^*^S^*^)^_3_Bz**)_4_(Zn^2+^)_3_ preserves the preformed propeller-shaped chirality (Scheme 3C). Notably, the suggested ternary complex was successfully identified by HR ESI MS, *m*/*z* calcd. as [(ImH^–^)_3_(**Im*^R^*_3_Bz**)_4_(Zn^2+^)_3_(OSO_2_CF_3_)_1_ + 2CH_3_CN]^2+^, 1440.51049; found 1440.51380 (Fig. 6). These results were obtained from an ESI mass spectrum of (**Im*^R^*_3_Bz**)_4_(Zn^2+^)_3_ in the presence of 3.0 equivalents of ImH_2_ in acetonitrile. The subsequently formed coordinatively saturated (ImH_2_)_3_(**Im^(^*^R^*^or^*^S^*^)^_3_Bz**)_4_(Zn^2+^)_3_ assembly contained no Zn^2+^ center capable of binding with the additional monodentate co-ligands (Fig. 7a). However, we observed a second inflection point in the CD titration plot (*vide supra*, Fig. 4 and 5 insets), which is associated with a sharp change of the CD spectrum (*vide supra*, Fig. 4 and 5, yellow lines). We posit that this feature arises from a supramolecular chirality transition driven by monodentate ligand binding (Scheme 3D). The observed intense CD signals (Fig. 4 and 5, yellow line) are characteristic of an exciton-coupled biphasic (splitting) CD pattern caused by twisting of the chromophore molecules.21c,^26^,^34^–^36^ In this context, Katoono *et al.* have recently developed stacked dimer assemblies of *C*_3_-symmetric benzene rings containing ethynyl spacers in the twisting positions.^26^ They provided CD profiles, which resemble those in our study (yellow lines in Fig. 4 and 5), suggesting structural similarities to our proposed stacked dimer assemblies here.^26^ From this analysis, we estimated the stacked dimer assemblies in the twisting position [(ImH_2_)_*m*_(**Im_3_Bz**)_2_(Zn^2+^)_3_] (*m* = 4–6) to be the most probable assembly in solution in the second stage of the titration (corresponding to yellow lines in Fig. 4 and 5). It should be noted that we have measured the ESI mass spectrum of **Im*^S^*_3_Bz** in the presence of Zn^2+^ and 1.7 equivalents (toward Zn^2+^) of ImH_2_, where the fragment mass of [(ImH_2_)_*m*_(**Im*^S^*_3_Bz**)_2_(Zn^2+^)_3_] (*m* = 4–6) was found (Fig. S10†).

In order to investigate whether the reversible change between the (**Im*^R^*_3_Bz**)_4_(Zn^2+^)_3_ and (ImH_2_)_*m*_(**Im*^R^*_3_Bz**)_2_(Zn^2+^)_3_ complexes (Scheme 3C and D) is possible or not, we have measured CD spectra of (ImH_2_)_*m*_(**Im*^R^*_3_Bz**)_2_(Zn^2+^)_3_ upon addition of Zn^2+^ that acts as the sequester of ImH_2_ in (ImH_2_)_*m*_(**Im*^R^*_3_Bz**)_2_(Zn^2+^)_3_ (Fig. S11†). The CD intensity due to (ImH_2_)_*m*_(**Im*^R^*_3_Bz**)_2_(Zn^2+^)_3_ decreased with increasing Zn^2+^ concentration (Fig. S11†), where the CD spectrum of (ImH_2_)_*m*_(**Im*^R^*_3_Bz**)_2_(Zn^2+^)_3_ converted to that of (ImH_2_)_3_(**Im*^R^*_3_Bz**)_4_(Zn^2+^)_3_ upon addition of 2.5 × 10^–5^ M of Zn^2+^ (Fig. S11†). Hence, conversion of (ImH_2_)_*m*_(**Im*^R^*_3_Bz**)_2_(Zn^2+^)_3_ to (ImH_2_)_3_(**Im*^R^*_3_Bz**)_4_(Zn^2+^)_3_ (Scheme 3D) is reversible. Further addition of Zn^2+^ to the resulting solution caused a subsequent CD spectral change (Fig. S11†), indicating a structural transition of (ImH_2_)_3_(**Im*^R^*_3_Bz**)_4_(Zn^2+^)_3_. However, the resulting CD spectrum is slightly different from that of Zn_3_(**Im*^R^*_3_Bz**)_4_ (Fig. 4, red line). Thus, the reversible change between (ImH_2_)_3_(**Im*^R^*_3_Bz**)_4_(Zn^2+^)_3_ and (**Im*^R^*_3_Bz**)_4_(Zn^2+^)_3_ (Scheme 3C) is not possible under these conditions.

The ligand-driven architectural transformation is further discussed in detail by NMR titration experiments. We used **Im_3_Bz** instead of **Im^(^*^R^*^or^*^S^*^)^_3_Bz** in this case, because of the NMR broadening observed for (**Im^(^*^R^*^or^*^S^*^)^_3_Bz**)_4_(Zn^2+^)_3_ (*vide supra*, Fig. S1†). Note that the NMR titration experiments with **Im*^R^*_3_Bz** give almost the same results as those obtained with **Im_3_Bz** except for the initial NMR broadening (Fig. S12†). Upon addition of 0–3.0 equivalents of ImH_2_ to the resulting (**Im_3_Bz**)_4_(Zn^2+^)_3_ assembly in CD_3_CN (Fig. 7d, red line to green line), the well-resolved NMR signals from (**Im_3_Bz**)_4_(Zn^2+^)_3_ (red line) disappeared with a concomitant rise of sharp peaks at 8.48, 7.97, 7.40, and 7.32 ppm (j, k, l, and m, respectively) and broad signals at around 7.32 and 7.03 ppm (E and F, respectively). Among them, the sharp peaks (j and k) are considered to originate from two coordinated species in solution, and the peak “k” disappears with a concomitant increase of the relative intensity of the peak “j” with increasing molar ratio (*x*) [ImH_2_]/[(**Im_3_Bz**)_4_(Zn^2+^)_3_]_0_ (Fig. 7d, green line to yellow line). Upon further increasing ImH_2_ concentrations, the remaining peak “j” gradually shifted upfield and became the same NMR signal of free **Im_3_Bz** owing to the central benzene proton (Fig. 7c red square) at a molar ratio of [ImH_2_]/[(**Im_3_Bz**)_4_(Zn^2+^)_3_]_0_ = 12.0 (Fig. 7d, yellow line to purple line). This observation is consistent with dissociation of the self-assemblies to yield the ImH_2_–Zn^2+^ complex at higher concentrations of ImH_2_ (Scheme 3E). Taken together, we conclude that the peak “j” originates from the central benzene protons of **Im_3_Bz** in the subsequently formed stacked dimer assembly [(ImH_2_)_*m*_(**Im_3_Bz**)_2_(Zn^2+^)_3_], and the peak “k” derives from (ImH_2_)_3_(**Im_3_Bz**)_4_(Zn^2+^)_3_ prior to the stacked dimer assembly formation. Partial dissociation and reassociation likely occur on the NMR timescale in both assemblies, giving rise to a rather simple NMR signal pattern (Fig. 7c, green line). The two assemblies coexist at a molar ratio of [ImH_2_]/[(**Im_3_Bz**)_4_(Zn^2+^)_3_]_0_ = 3.0 (Fig. 7d, green line) under the conditions used for the NMR experiments.^37^ Imidazole protons (as well as CH_2_ protons from the side chains) of **Im_3_Bz** overlapped at similar chemical shifts for the two assemblies and showed rather broad signals (E and F on the green line).^38^ Conversely, the chemical shifts of the central benzene protons were markedly different between the two assemblies (peaks j and k), where the peak “j” shifted downfield compared with the peak “k” that is assigned to the propeller-shaped assembly (Fig. 7d). This differentiation may be the result of a difference in the relative positional relationship of the central benzene rings between the two assemblies. In the propeller-shaped assembly (Fig. 7a), the central *C*_3_-symmetric ligand (red colored molecule) is almost perpendicular to the three propeller-shaped ligands (blue colored molecules). Hence, their chemical environments are not markedly different from those of the free ligand. Conversely, in the stacked dimer assembly, clipping by the three Zn^2+^ ions forces the two benzene rings into a twisted face-to-face position (Fig. 7b). The observed downfield shift (peak j in Fig. 7d) is attributed to the deshielding effect of the stacked benzene rings, which gives experimental confirmation of the stacked dimer assembly.^39^,^40^

**Fig. 8:**
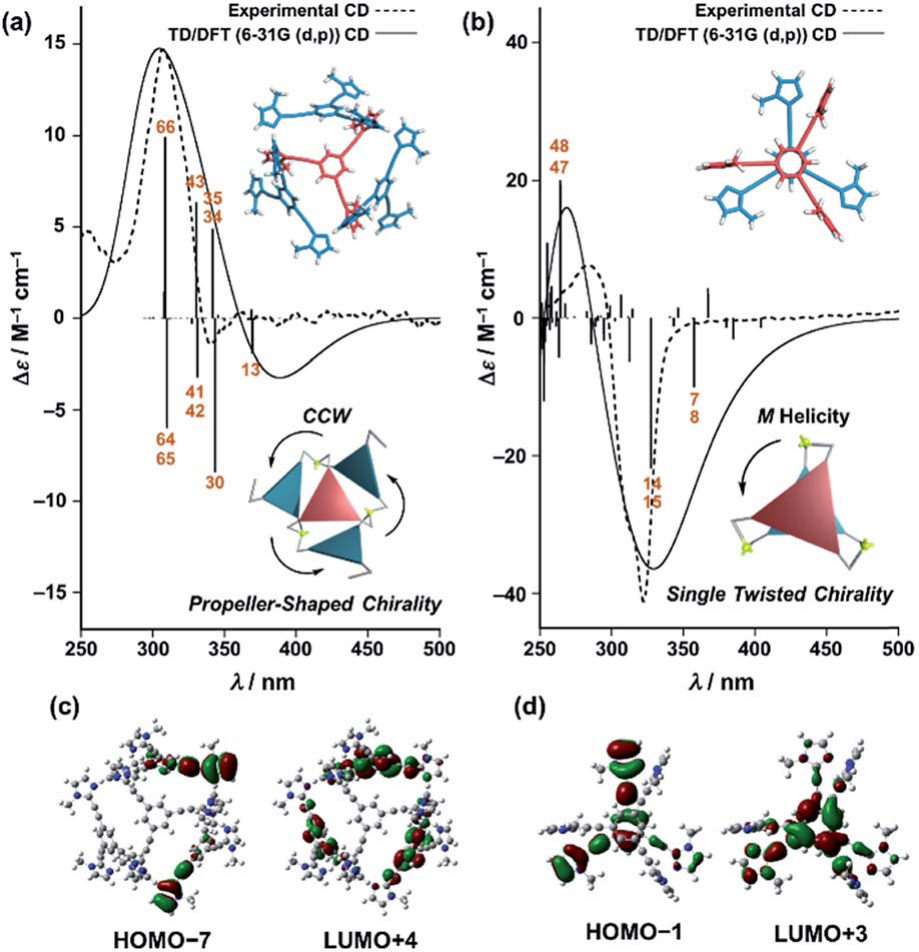
Dashed lines show experimental CD spectra of (a) (**Im*^R^*_3_Bz**)_4_(Zn^2+^)_3_ (corresponding to the red line in Fig. 4) and (b) (ImH_2_)_*m*_(**Im*^S^*_3_Bz**)_2_(Zn^2+^)_3_ (corresponding to the yellow line in Fig. 5). Solid lines show theoretical CD spectra [TD-DFT/B3LYP-6-31G(d,p)] of the *C*_3_-symmetric ligands in (a) the propeller-shaped arrangement and (b) twisting dimer arrangement. Calculated spectrum in (a) is vertically scaled (scaling factor = 0.85). The numbered excitations correspond to those with high rotatory strength. Two molecular orbitals involved in (c) excitation 43 (17%) in (a) and (d) excitation 14 (47%) in (b). Insets: schematic representation of (a) propeller-shaped chirality and (b) single twisted chirality.

Finally, we performed time-dependent density functional theory (TD-DFT) calculations on the *C*_3_-symmetric ligands in a propeller-shaped arrangement and a twisting dimer arrangement (solid lines in Fig. 8a and b, respectively). Here, only electronic transitions of the *C*_3_-symmetric ligand part were calculated. Neither the Zn^2+^ (*d*^10^ configuration) nor the imidazole co-ligands have any appreciable absorption above 250 nm contributing to the observed CD spectra (dashed lines in Fig. 8). In addition, the chiral alkyl side chains of the *C*_3_-symmetric ligands were replaced by methyl groups to reduce the calculation complexity. These modifications enabled us to perform the TD-DFT calculations in a reasonable time frame. The theoretical CD spectrum for the propeller-shaped geometry agreed well with the experimental one for (**Im*^R^*_3_Bz**)_4_(Zn^2+^)_3_ (Fig. 8a, solid line *vs.* dashed line), suggesting a counter clockwise (ccw) rotation for (**Im*^R^*_3_Bz**)_4_(Zn^2+^)_3_, and a clockwise (cw) rotation for (**Im*^S^*_3_Bz**)_4_(Zn^2+^)_3_. According to the calculated rotatory strength (see Fig. S13†), most of the high rotatory strength arises from electronic transitions within the three molecules that represent the propeller shape (blue colored molecules in the inset of Fig. 8a). For example, the dominant contribution (17%) of excitation 43 (Fig. 8a) is a transition from the HOMO–7 to LUMO+4, which features molecular orbitals delocalized over the two and three molecules in the propeller-shaped arrangement, respectively (Fig. 8c). Conversely, the HOMO, HOMO–1, and HOMO–2 are localized on the central *C*_3_-symmetric ligand (red colored molecule in the inset of Fig. 8a, see Fig. S13†). Those higher energy levels do not contribute to a high rotatory strength in Fig. 8a (see Fig. S13†). Consequently, the propeller-shaped assembly (**Im*^R^*_3_Bz**)_4_(Zn^2+^)_3_ has a positive Cotton effect at shorter wavelengths and the extremely weak negative Cotton effect at the longer wavelength is negligible (Fig. 8a). In contrast, splitting of the CD pattern is found in both the experimental and theoretical CD spectra for the stacked dimer in the twisting position (Fig. 8b), where both spectra show a strong negative Cotton effect in the first Cotton band and a weak positive Cotton effect in the second Cotton band. These calculations suggest that most of the high rotatory strength arises from electronic transitions between the two molecules in the twisting position (Fig. S14†). For example, a transition from HOMO–1 to LUMO+3 is the dominant contribution (47%) of excitation 14 in Fig. 8b, in which the molecular orbital of LUMO+3 is essentially delocalized over the two central benzene rings (Fig. 8d). This calculation indicates an *M* helix (excess) for (ImH_2_)_*m*_(**Im*^S^*_3_Bz**)_2_(Zn^2+^)_3_ and a *P* helix (excess) for (ImH_2_)_*m*_(**Im*^R^*_3_Bz**)_2_(Zn^2+^)_3_.

## Conclusions

We have successfully demonstrated a supramolecular chirality transition driven by monodentate ligand binding. Newly synthesized *C*_3_-symmetric ligands (**Im^(^*^R^*^or^*^S^*^)^_3_Bz**) containing three chiral imidazole side arms (Im^(*R* or *S*)^) with ethynyl spacers assemble into a propeller-shaped assembly [(**Im^(^*^R^*^or^*^S^*^)^_3_Bz**)_4_(Zn^2+^)_3_] with 0.75 equivalents of Zn^2+^ in acetonitrile in the absence of monodentate co-ligands (imidazole: ImH_2_). The resulting (**Im^(^*^R^*^or^*^S^*^)^_3_Bz**)_4_(Zn^2+^)_3_ assembly is formally coordinatively unsaturated (coordination number: *n* = 3) and therefore capable of accepting a total of three equivalents of monodentate co-ligands (ImH_2_) to yield a coordinatively saturated assembly. The resulting coordinatively saturated assembly [(ImH_2_)_3_(**Im^(^*^R^*^or^*^S^*^)^_3_Bz**)_4_(Zn^2+^)_3_] preserves the preformed propeller-shaped chirality of (**Im^(^*^R^*^or^*^S^*^)^_3_Bz**)_4_(Zn^2+^)_3_. However, addition of monodentate co-ligands transforms the supramolecular chirality from propeller-shaped chirality into single-twist chirality, when (ImH_2_)_3_(**Im^(^*^R^*^or^*^S^*^)^_3_Bz**)_4_(Zn^2+^)_3_ transforms into a stacked dimer assembly (ImH_2_)_*m*_(**Im^(^*^R^*^or^*^S^*^)^_3_Bz**)_2_(Zn^2+^)_3_ (*m* = 4–6). The present strategy shows promise for the rational design of dynamic coordination chirality capable of alternating between chiral objects of different shapes driven by a specific external stimulus.

## Conflicts of interest

There are no conflicts to declare.

## Supplementary Material

Supplementary information
