# Supplementary material for: Supramolecular chirality transformation driven by monodentate ligand binding to a coordinatively unsaturated self-assembly based on C3-symmetric ligands
Source: Chem Sci. 2019 Mar 5;10(15):4236–45. doi: 10.1039/c9sc00399a (PMC6471804; doi:10.1039/c9sc00399a)
Supplement: Supplementary file 1 [file SC-010-C9SC00399A-s001.pdf]

Electronic Supplementary Material (ESI) for Chemical Science.
This journal is © The Royal Society of Chemistry 2019

Supporting Information

Supramolecular chirality transformation driven by monodentate ligand binding to coordinatively unsaturated self-assembly based on C_3-symmetric ligands

Yuki Imai and Junpei Yuasa*

Graduate School of Chemical Science and Technology, Tokyo University of Science, 1-3 Kagurazaka, Shinjuku-ku, Tokyo 162-8601, Japan.

* To whom correspondence should be addressed.

E-mail: yuasaj@rs.tus.ac.jp

Experimental Section

General. Chemicals were purchased from Wako Pure Chemical Industries Ltd. and used as received without further purification. 1,3,5-tris((trimethylsilyl)ethynyl)benzene, (*R*)- and (*S*)-2-iodo-1-(2-methoxypropyl)-1*H*-imidazole and 1-ethyl-2-iodo-1*H*-imidazole were prepared according to a procedure described previously.[1,2] ^{1}H and ^{13}C NMR spectra were measured with JEOL, JNM-ECZ400S (400 MHz). UV-vis absorption spectra were measured at ambient temperature using JASCO V-660. Mass spectra were measured with mass spectrometers (JEOL AccuTOF JMS-T100CS). CD spectra were measured by JASCO J-820 Spectropolarimeter. TD-DFT calculation was conducted using Gaussian 09.

Synthesis

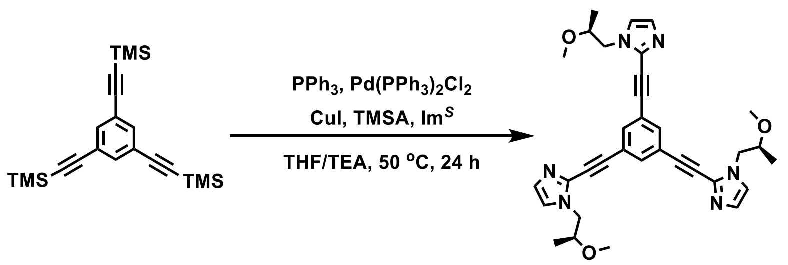

Preparation of 1,3,5-tris((1-((*S*)-2-methoxypropyl)-1*H*-imidazol-2-yl)ethynyl)benzene: To a flame dried 2-necked flask containing CuI (16 mg, 0.08 mmol), 1,3,5-tris((trimethylsilyl)ethynyl)benzene (300 mg, 0.8 mmol), PPh_3 (22 mg, 0.08 mmol), (*S*)-2-iodo-1-(2-methoxypropyl)-1*H*-imidazole (870 mg, 3 mmol), degassed THF (10 mL), and degassed triethylamine (10 mL) were added. Then $Pd(PPh_3)_2Cl_2$ (58 mg, 0.08 mmol) and TBAF (1M in THF, 4 mL) were added. This solution was stirred at 50 °C for 1 day. The reaction mixture was filtered, and the filtrate was evaporated. The crude product was dissolved in chloroform and washed with saturated aqueous ammonium chloride. The organic layer was dried over anhydrous sodium sulfate. After evaporation, the crude product was purified by column chromatography on silica gel (chloroform/methanol = 9/1) and GPC with chloroform to give 1,3,5-tris((1-((*S*)-2-methoxypropyl)-1*H*-imidazol-2-yl)ethynyl)benzene as pale yellow oil (30 mg, 9%). ^{1}H NMR (500 MHz, $CDCl_3$): δ 7.66 (s, 3H), 7.13 (s, 3H), 7.10 (s, 3H), 4.07–4.18 (m, 6H), 3.65–3.71 (m, 3H), 3.34 (s, 9H), 1.20 (d, $J = 6.3$ Hz, 9H). ^{13}C NMR (125 MHz, $CDCl_3$) δ 134.30, 131.36, 129.95, 123.18, 122.11, 90.33, 80.61, 76.04, 56.77, 51.79, 16.81. HRMS (ESI+): *m*/*z* calcd. $C_{33}H_{36}N_6NaO_3$ $[M+Na]^+$: 587.27466; found 587.27467.

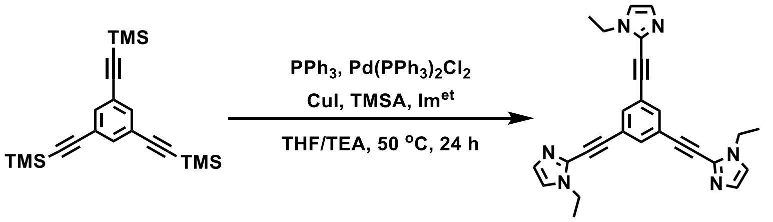

Preparation of 1,3,5-tris((1-ethyl-1*H*-imidazol-2-yl)ethynyl)benzene: To a flame dried 2-necked flask containing CuI (10 mg, 0.05 mmol), 1,3,5-tris((trimethylsilyl)ethynyl)benzene (300 mg, 0.8 mmol), PPh_3 (13 mg, 0.05 mmol), 1-ethyl-2-iodo-1*H*-imidazole (726 mg, 3.3 mmol), degassed THF (10 mL), and degassed triethylamine (10 mL) were added. Then $Pd(PPh_3)_2Cl_2$ (34 mg, 0.05 mmol) and TBAF (1M in THF, 3.3 mL) were added. This solution was stirred at 50 °C for 1 day. The reaction mixture was filtered, and the filtrate was evaporated. The crude product was dissolved in chloroform and washed with saturated aqueous ammonium chloride. The organic layer was dried over anhydrous sodium sulfate. After evaporation, the crude product was purified by column chromatography on silica gel (chloroform/methanol = 9/1) and GPC with chloroform to give 1,3,5-tris((1-ethyl-1*H*-imidazol-2-yl)ethynyl)benzene as off white needle crystal (50 mg, 14%). ^{1}H NMR (500 MHz, $CDCl_3$): δ 7.70 (s, 3H), 7.13 (d, J = 1.1 Hz, 3H), 7.02 (d, J = 1.1 Hz, 3H), 4.17 (q, J = 7.3 Hz, 6H), 1.51 (t, J = 7.4 Hz, 9H). ^{13}C NMR (125 MHz, $CDCl_3$) δ 134.31, 130.82, 130.15, 123.15, 120.00, 90.40, 80.38, 42.00, 16.09. HRMS (ESI+): *m*/*z* calcd. $C_{27}H_{25}N_6$ $[M+H]^+$: 433.21407; found 433.21346.

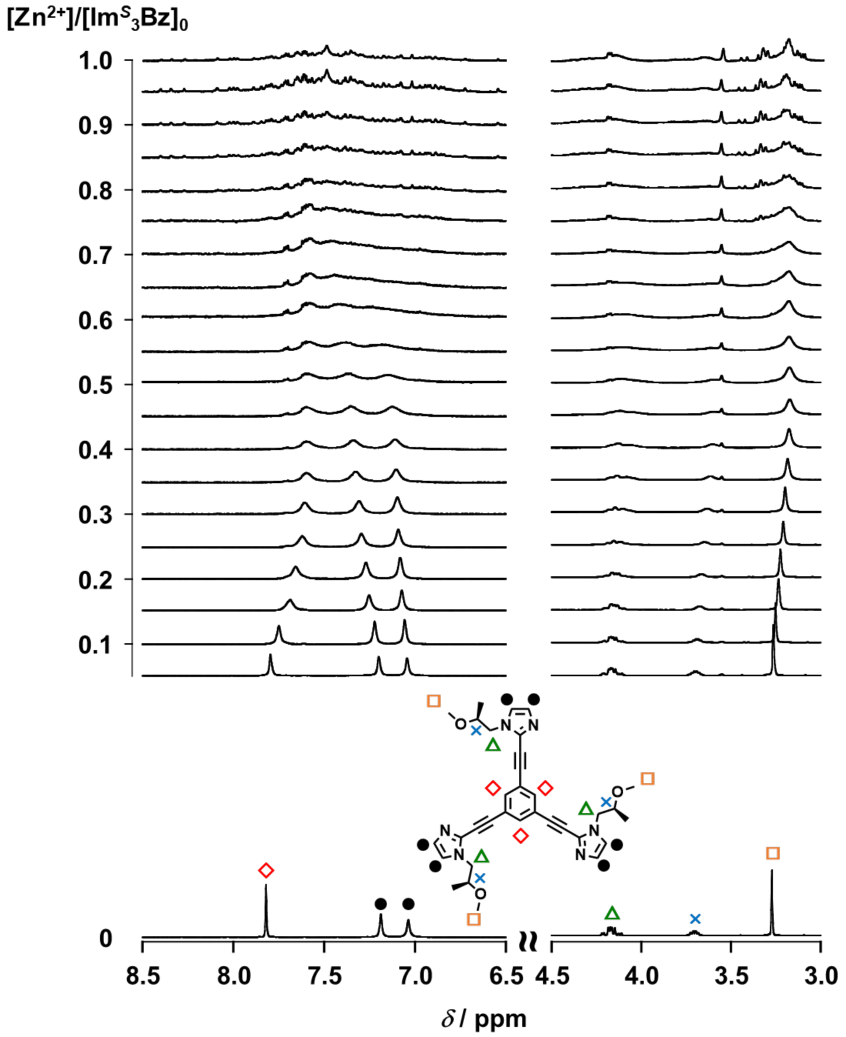

Fig. S1 Stacked ^{1}H NMR spectra of $Im^S{}_3Bz$ (1.9×10^{-3} M) in the presence of Zn^{2+} ($0–1.9 \times 10^{-3}$ M) in CD_3CN at 298 K.

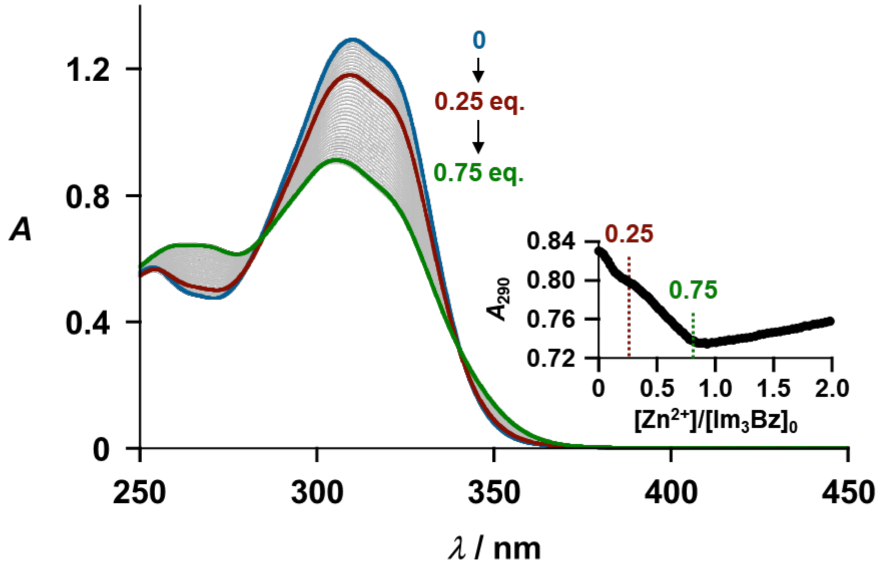

Fig. S2 UV/Vis absorption spectra of Im_3Bz (2.0×10^{-5} M) in the presence of Zn^{2+} (0 (blue)–5.0×10^{-6} (red)–1.5×10^{-5} M (green)) in acetonitrile at 298 K.

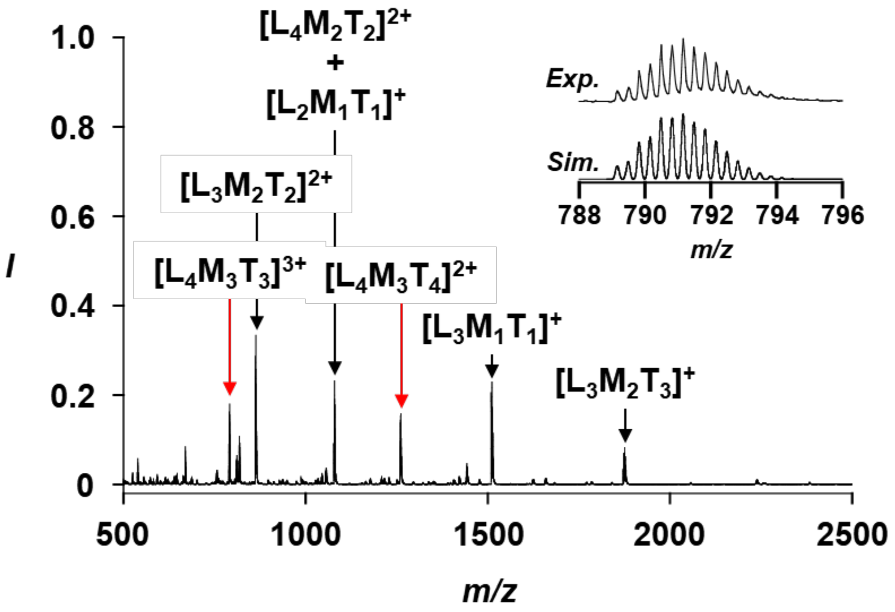

Fig. S3 Positive ESI-MS spectrum of Im_3Bz (normalized by their most intense fragment at *m/z* = 432.2 due to the free ligand) in acetonitrile (2.0×10^{-3} M) with the presence of Zn^{2+} (1.5×10^{-4} M). Inset: Isotopically resolved signals at *m/z* = 789.2 and the calculated isotopic distributions for $[(Im_3Bz)_4(Zn^{2+})_3(OSO_2CF_3)_3]^{3+}$. Objects correspond to the mass peak assignment (L: Im_3Bz, M: Zn^{2+}, T: $OSO_2CF_3^-$).

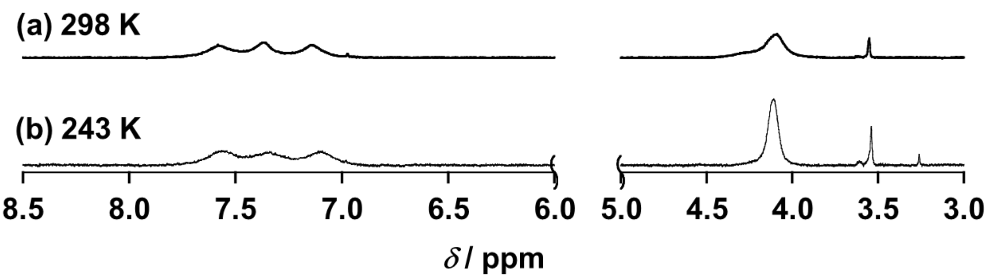

Fig. S4 ^{1}H NMR spectra of Im_3Bz (1.6×10^{-3} M) in the presence of Zn^{2+} (8.0×10^{-4} M) in CD_3CN at (a) 298 and (b) 243 K.

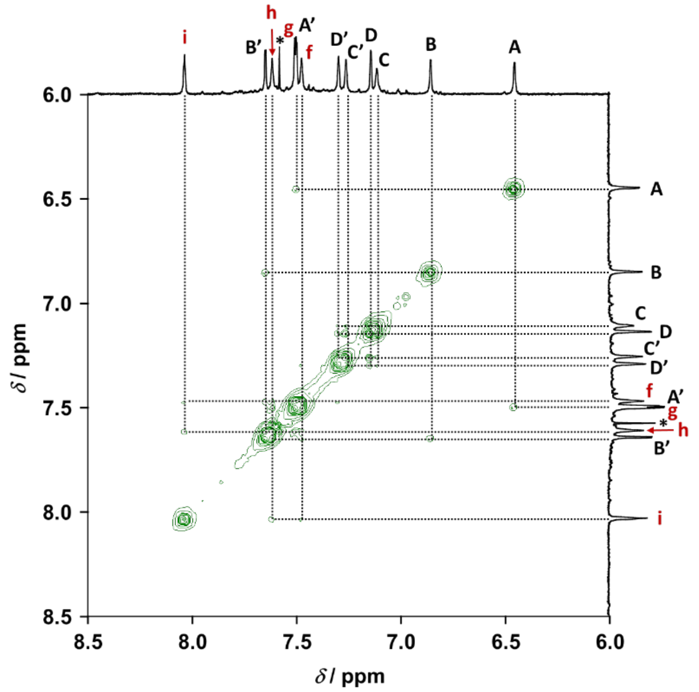

Fig. S5 ^{1}H-^{1}H COSY NMR of Im_3Bz (1.76×10^{-3} M) in the presence of Zn^{2+} (1.32×10^{-3} M) in CD_3CN at 6.0–8.5 ppm. Asterisk denotes the solvent peak ($CHCl_3$).

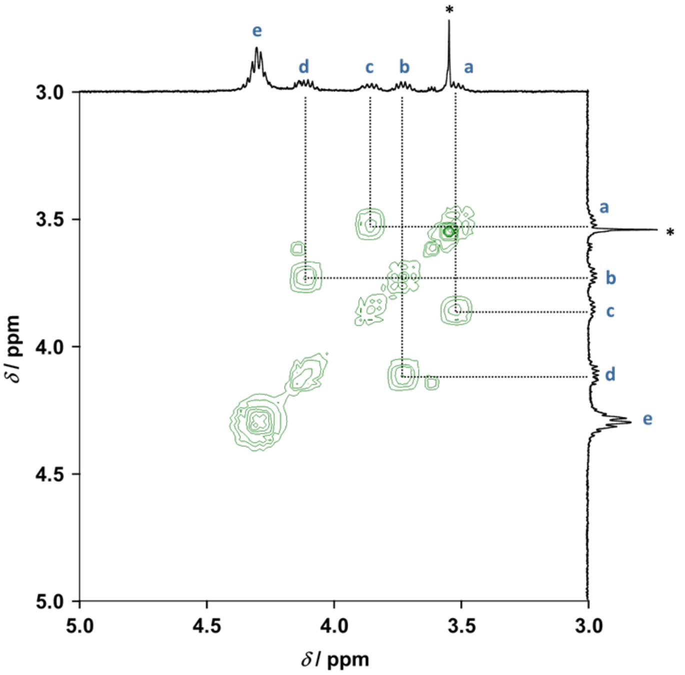

Fig. S6 ^{1}H-^{1}H COSY NMR of Im_3Bz (1.76×10^{-3} M) in the presence of Zn^{2+} (1.32×10^{-3} M) in CD_3CN at 3.0–5.0 ppm.

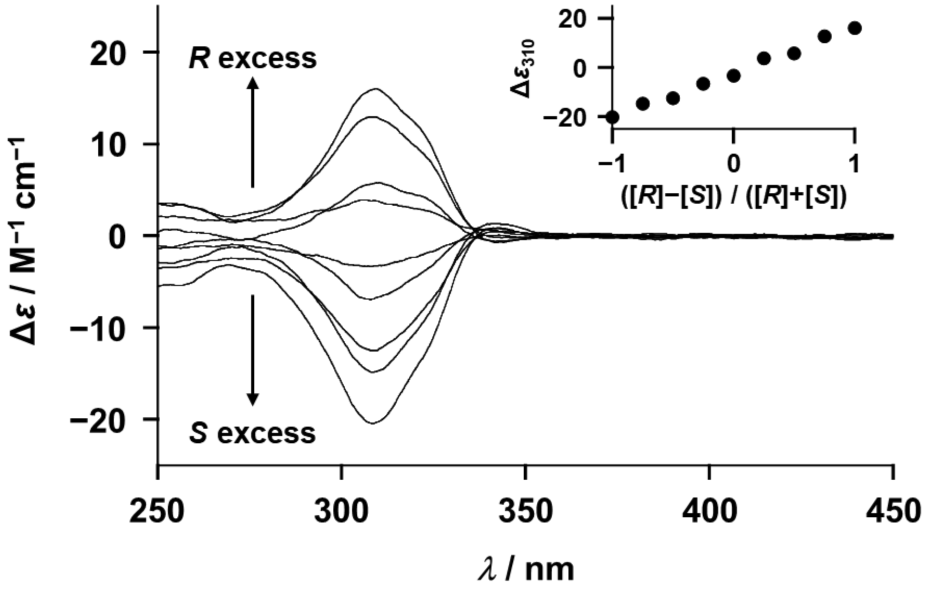

Fig. S7 CD spectra of different enantiomeric excess ratio of $Im^R{}_3Bz$ and $Im^S{}_3Bz$ in the presence of Zn^{2+} (1 equiv.) in acetonitrile.

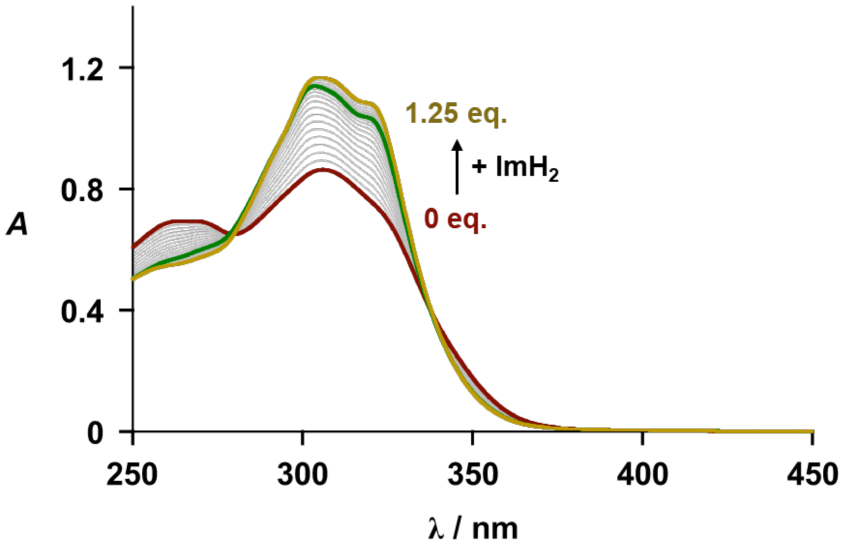

Fig. S8 UV-Vis absorption spectral changes observed upon titration of an acetonitrile solution of $Im^{R}{}_3Bz$ (2.0×10^{-5} M) containing Zn^{2+} (1.5×10^{-5} M) by ImH_2 ($0–6.0 \times 10^{-5}$ M, 0: red line, 1.5×10^{-5} M: green line, 2.5×10^{-5} M: yellow line).

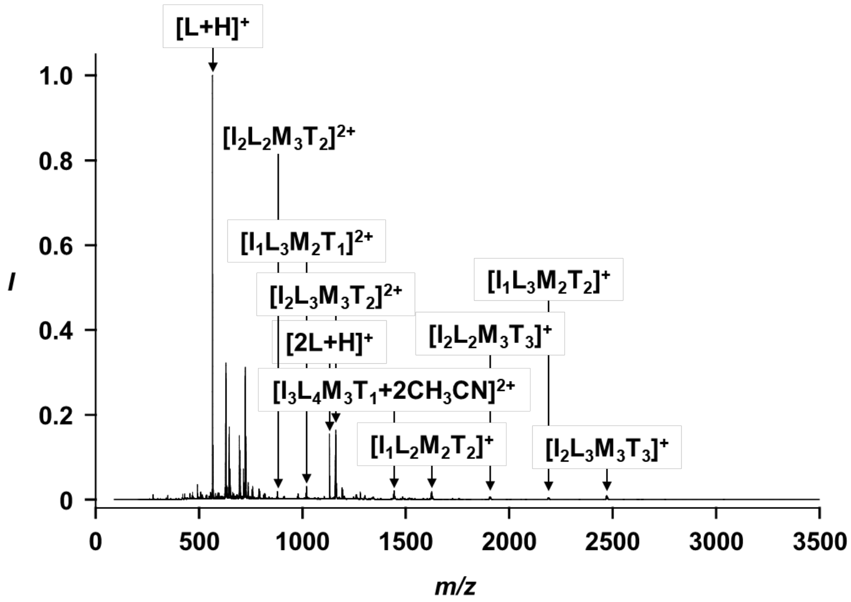

Fig. S9 Positive ESI-MS spectrum of $Im^{S}{}_3Bz$ (normalized by their most intense fragment at m/z = 564.3 due to the free ligand) in acetonitrile (2.2×10^{-3} M) with the presence of Zn^{2+} (1.7×10^{-3} M) and ImH_2 (1.7×10^{-3} M). Objects correspond to the mass peak assignment (I: ImH^{-}, L: $Im^{S}{}_3Bz$, M: Zn^{2+}, T: $OSO_2CF_3{}^{-}$).

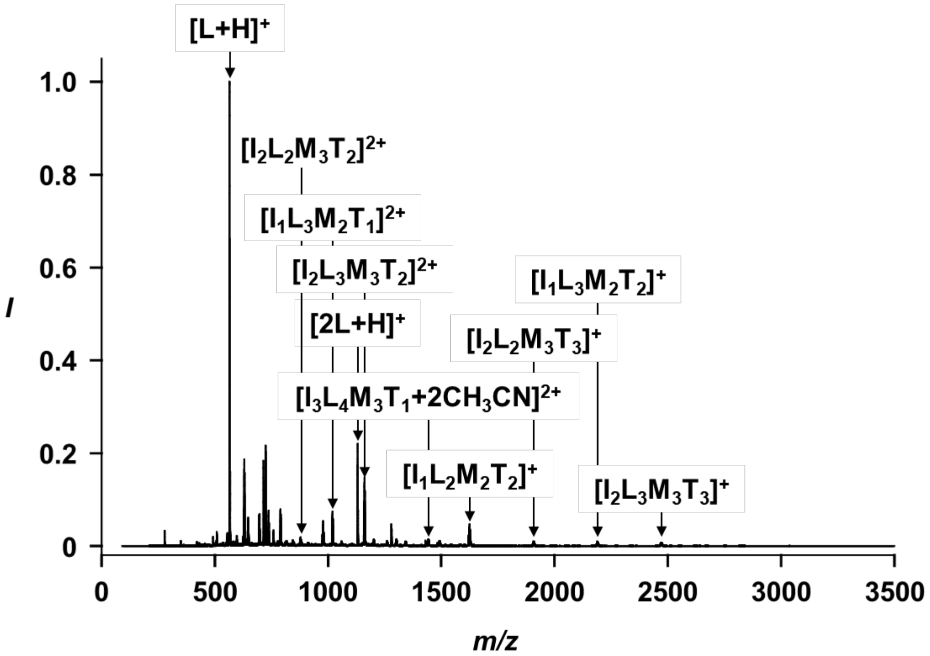

Fig. S10 Positive ESI-MS spectrum of $Im^{S}{}_3Bz$ (normalized by their most intense fragment at *m/z* = 564.3 due to the free ligand) in acetonitrile (2.2×10^{-3} M) with the presence of Zn^{2+} (1.7×10^{-3} M) and ImH_2 (2.8×10^{-3} M). Objects correspond to the mass peak assignment (I: ImH^-, L: $Im^{S}{}_3Bz$, M: Zn^{2+}, T: $OSO_2CF_3{}^-$).

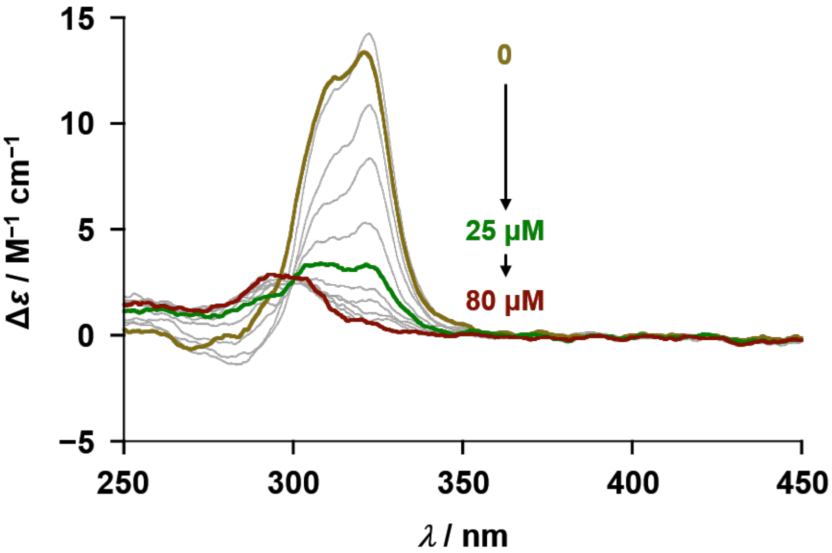

Fig. S11 CD spectral changes observed upon addition of Zn^{2+} (0 (yellow)–2.5×10^{-5} (green)– 8.0×10^{-5} M (red)) to an acetonitrile solution of $(ImH_2)_m$(**Im$^{R}{}_3$Bz**)$_2(Zn^{2+})_3$. The initial $(ImH_2)_m$(**Im$^{R}{}_3$Bz**)$_2(Zn^{2+})_3$ complex was prepared by $Im^{R}{}_3Bz$ (2.0×10^{-5} M), Zn^{2+} (2.0×10^{-5} M) and imidazole (2.0×10^{-5} M) in situ.

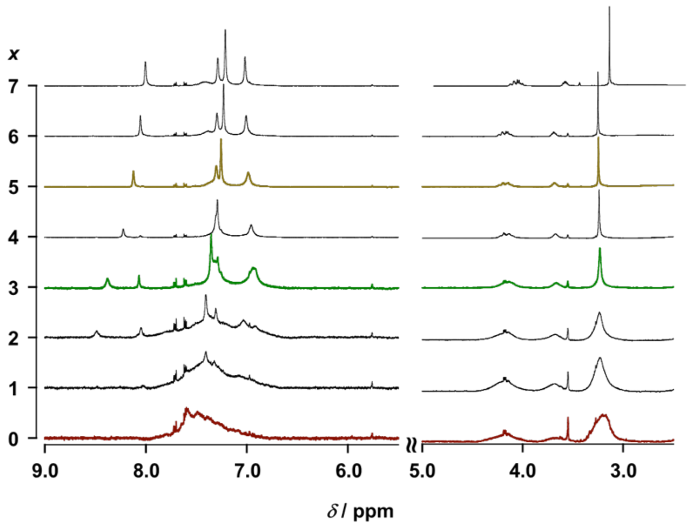

Fig. S12 Stacked ^{1}H NMR spectra of $Im^{R}{}_3Bz$ (2.0×10^{-3} M) with Zn^{2+} (1.5×10^{-3} M) in the presence of ImH_2 (0–3.5×10^{-3} M, 0: red line, 1.5×10^{-3} M: green line, 2.5×10^{-3} M: yellow line) in CD_3CN at 298 K (x = $[ImH_2]/[(Im^{R}{}_3Bz)_4(Zn^{2+})_3]_0$).

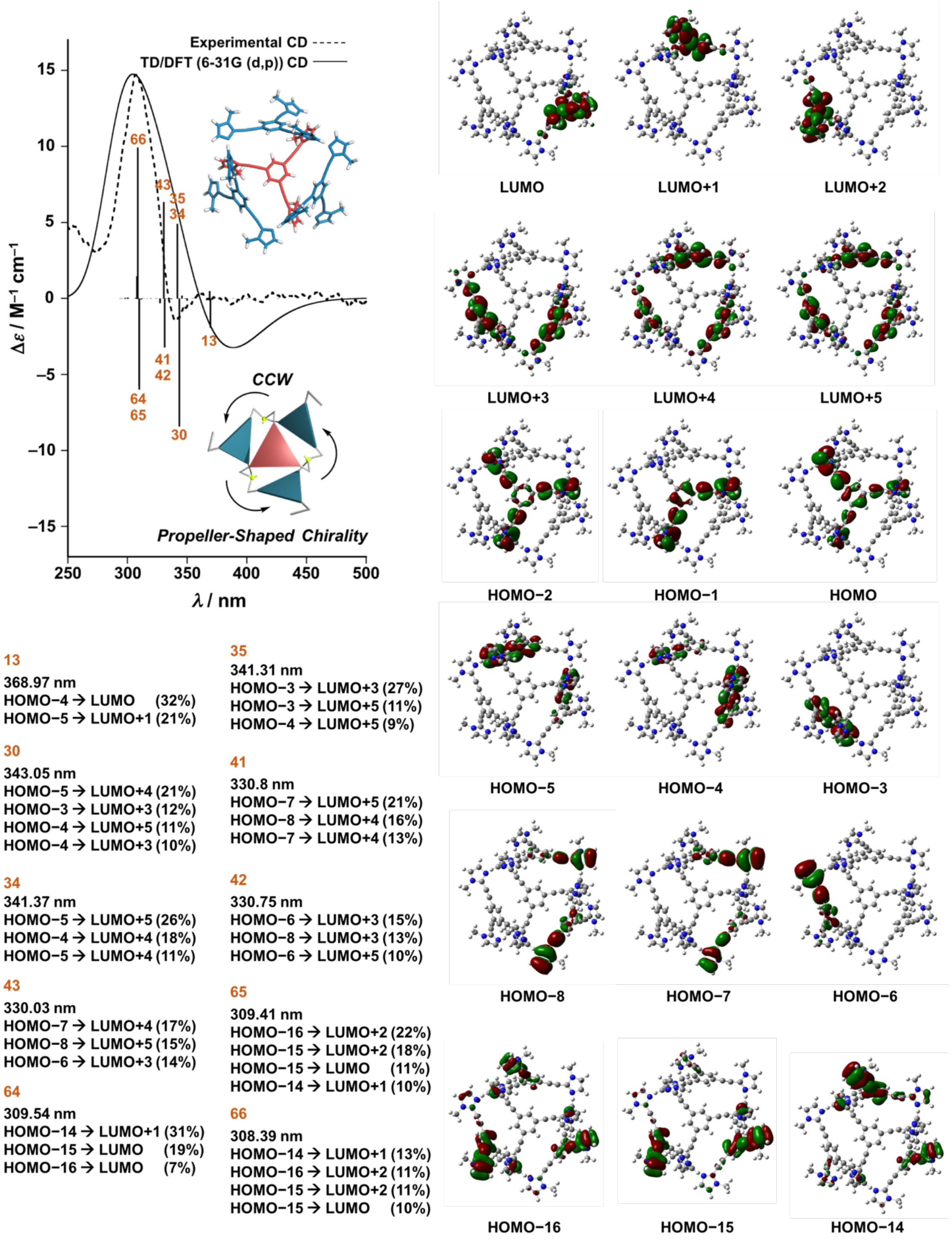

Fig. S13 Assignment of rotatory strength for C_3-symetric ligands in the propeller-shaped arrangement.

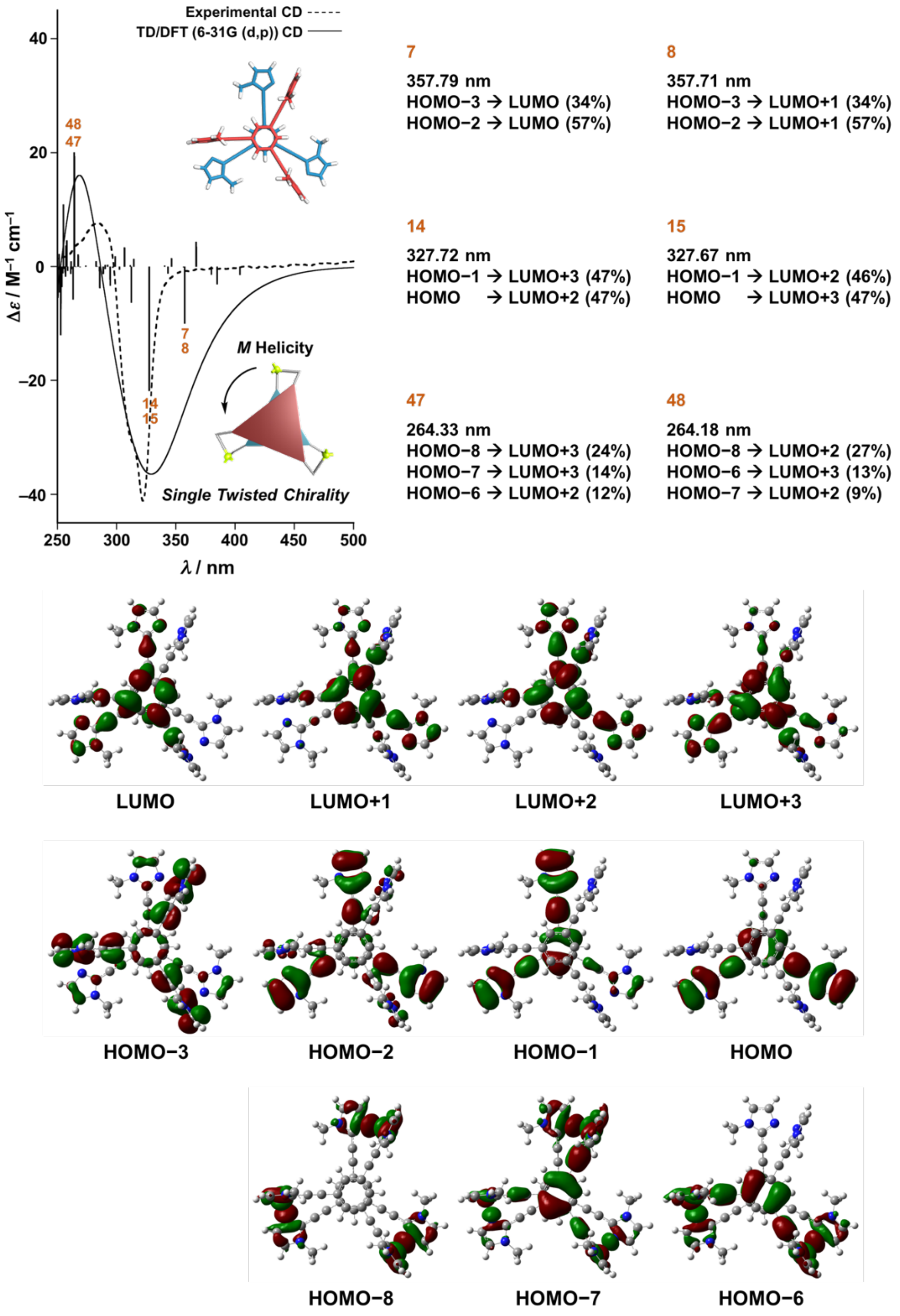

Fig. S14 Assignment of rotatory strength for C_3-symetric ligands in the twisting dimer arrangement.

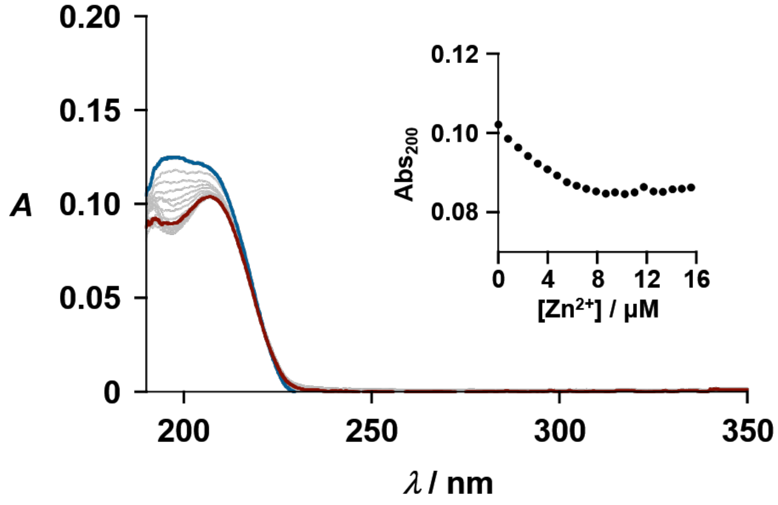

Fig. S15 UV-vis absorption spectra of imidazole (2.0×10^{-5} M) in the presence of Zn^{2+} (0 (blue)–1.0×10^{-5} (red)) in acetonitrile.

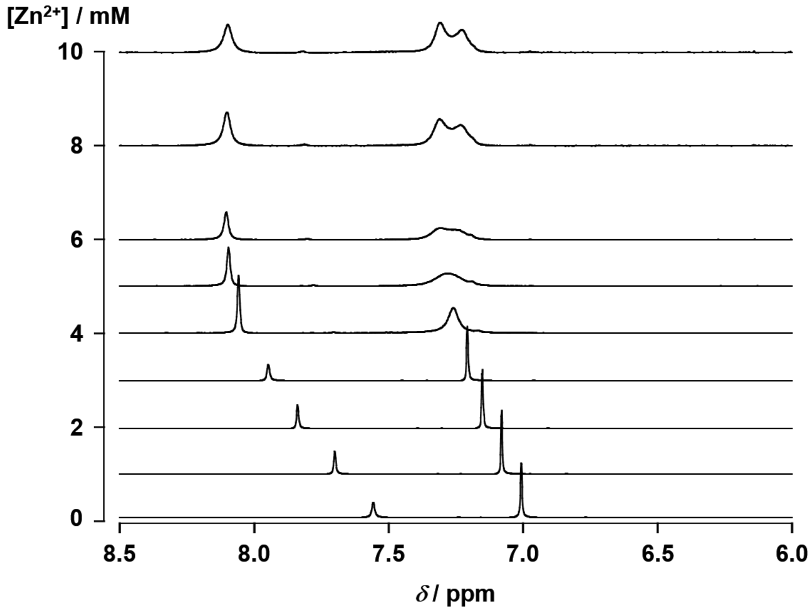

Fig. S16 ^{1}H NMR spectra of imidazole (1.0×10^{-2} M) in the presence of Zn^{2+} (0–1.0×10^{-2} M) in CD_3CN.

References

(1) Chen, Z.; Chen, M.; Yu, Y.; Wu, L. *Chem. Commun.* **2017**, *53*, 1989–1992.

(2) Imai, Y.; Nakano, Y.; Kawai, T.; Yuasa, J. *Angew. Chem. Int. Ed.* **2018**, *57*, 8973–8978.
